# Clinical features and molecular landscape of cuproptosis signature‐related molecular subtype in gastric cancer

**DOI:** 10.1002/imt2.190

**Published:** 2024-04-05

**Authors:** Wei Chong, Huicheng Ren, Hao Chen, Kang Xu, Xingyu Zhu, Yuan Liu, Yaodong Sang, Han Li, Jin Liu, Chunshui Ye, Liang Shang, Changqing Jing, Leping Li

**Affiliations:** ^1^ Department of Gastrointestinal Surgery Shandong Provincial Hospital Affiliated to Shandong First Medical University Jinan China; ^2^ Key Laboratory of Engineering of Shandong Province, Shandong Provincial Hospital, Medical Science and Technology Innovation Center Shandong First Medical University & Shandong Academy of Medical Sciences Jinan China; ^3^ Department of Gastrointestinal Surgery Zibo Central Hospital Zibo China; ^4^ Clinical Research Center of Shandong University, Clinical Epidemiology Unit Qilu Hospital of Shandong University Jinan China; ^5^ Department of Gastroenterological Surgery The First Affiliated Hospital of Shandong First Medical University Jinan China; ^6^ Department of Gastroenterology Shandong Provincial Hospital Affiliated to Shandong First Medical University Jinan China

**Keywords:** clinical features, cuproptosis, gastric cancer, molecular landscape, prognosis model

## Abstract

Recent studies have highlighted the biological significance of cuproptosis in disease occurrence and development. However, it remains unclear whether cuproptosis signaling also has potential impacts on tumor initiation and prognosis of gastric cancer (GC). In this study, 16 cuproptosis‐related genes (CRGs) transcriptional profiles were harnessed to perform the regularized latent variable model‐based clustering in GC. A cuproptosis signature risk scoring (CSRS) scheme, based on a weighted sum of principle components of the CRGs, was used to evaluate the prognosis and risk of individual tumors of GC. Four distinct cuproptosis signature‐based clusters, characterized by differential expression patterns of CRGs, were identified among 1136 GC samples across three independent databases. The four clusters were also associated with different clinical outcomes and tumor immune contexture. Based on the CSRS, GC patients can be divided into CSRS‐High and CSRS‐Low subtypes. We found that *DBT, MTF1*, and *ATP7A* were significantly elevated in the CSRS‐High subtype, while *SLC31A1, GCSH, LIAS, DLAT, FDX1, DLD*, and *PDHA1* were increased in the CSRS‐Low subtype. Patients with CSRS‐Low score were characterized by prolonged survival time. Further analysis indicated that CSRS‐Low score also correlated with greater tumor mutation burden (TMB) and higher mutation rates of significantly mutated genes (SMG) in GC. In addition, the CSRS‐High subtype harbored more significantly amplified focal regions related to tumorigenesis (*3q27.1, 12p12.1, 11q13.3*, etc.) than the CSRS‐Low tumors. Drug sensitivity analyses revealed the potential compounds for the treatment of gastric cancer with CSRS‐High score, which were experimentally validated using GC cells. This study highlights that cuproptosis signature‐based subtyping is significantly associated with different clinical features and molecular landscape of GC. Quantitative evaluation of the CSRS of individual tumors will strengthen our understanding of the occurrence and development of cuproptosis and the treatment progress of GC.

## Highlights

- Four distinct cuproptosis signature‐based clusters are associated with different clinical outcomes and biological pathways and are highly consistent with distinct tumor immune contextures, respectively.
- Based on the cuproptosis signature risk score, GC patients with a higher CSRS score were characterized by decreased survival time and correlated with tumor adhesion state and lower tumor mutation loads.
- *DBT, MTF1*, or *ATP7A* were significantly elevated in the CSRS‐High subtype, while *ATP7B, SLC31A1, GCSH, LIAS, DLAT, FDX1, DLD*, and *PDHA1* were increased in the CSRS‐Low subtype.
- Drug sensitivity analyses revealed potential therapeutic compounds for GC with high CSRS scores.

## Graphical abstract

Four distinct cuproptosis signature‐based clusters of gastric cancer (GC) were identified based on the expression pattern of curated cuproptosis genes, and associated with different clinical outcomes, biological pathways and highly consistent with distinct tumor immune contexture respectively. Based on the cuproptosis signature risk score (CSRS), GC patients with higher CSRS scores were characterized by decreased survival time and correlated with tumor adhesion state and lower tumor mutation loads. *DBT, MTF1*, or *ATP7A* were significantly elevated in the CSRS‐High subtype, while *ATP7B, SLC31A1, GCSH, LIAS, DLAT, FDX1, DLD*, and *PDHA1* were significantly enriched in the CSRS‐Low subtype. Drug sensitivity analyses also revealed potential cancer compounds for GC with CSRS‐High scores, and molecular biology experiments were validated.

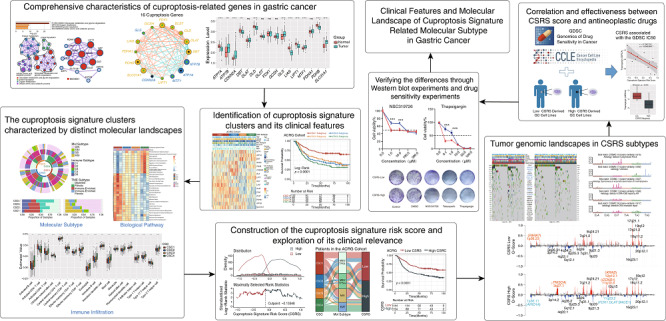

## INTRODUCTION

Gastric cancer (GC) is one of the most important cancers in the world, with the fifth incidence rate and the fourth mortality rate of the global world. The incidence rate is highest in east Asia and Eastern Europe [1]. The risk factors for gastric cancer prognosis include helicobacter pylori infection, age, tumor stage, subtype, and so forth [2]. With the increase of the aging population, the number of new cases of gastric cancer will continue to increase in the foreseeable future. In recent years, attempts have been made to combine the molecular types of gastric cancer with histological phenotypes and clinical features to understand the mechanism of occurrence and development of gastric cancer and explore important indicators to guide clinical decision making [3, 4, 5]. The exploitation and identification of new biomarkers have promoted the development of systemic therapy and targeted therapy [6, 7, 8]. At present, the primary treatment regimen of gastric cancer is surgical resection combined with perioperative adjuvant therapy [9]. There is growing appreciation that the GC patients with programmed death ligand 1 (PD‐L1) positive would benefit from the immune checkpoint inhibitor therapy [10]. Moreover, recent studies reported that vascular endothelial growth factor (VEGF) receptor inhibitor as a single agent or in combination with chemotherapy have improved the patients' survival outcomes [11]. At present, the early predictive screening and postoperative monitoring markers of gastric cancer are not clear. Prognosis biomarkers should be developed and utilized for the stratification of high‐risk population of gastric cancer to guide the therapy and intervention of gastric cancer. The personalized precision medicine services for patients using big data technology and standardized statistical models [12, 13].

Copper, an indispensable trace element for the human body, can exert cytotoxic effects and trigger programmed cell death when present in excessive amounts [14]. Previous studies have shown that both copper ionophores (DSF, etc.) and copper chelators (TTM, etc.) are considered to have anticancer effects [15, 16, 17]. Recent research indicates that copper ionophores trigger a particular type of controlled cellular demise, one that operates through mechanisms distinct from those of other forms of programmed cell death, such as apoptosis, pyroptosis, necrosis, and ferroptosis. [18]. Copper‐dependent cell death arises from the direct interaction of copper with lipoylated components of the tricarboxylic acid (TCA) cycle [18]. This interaction results in the aggregation of lipoylated proteins and the subsequent depletion of iron‐sulfur cluster proteins, ultimately leading to proteotoxic stress and the induction of cell death [18]. Two groups, positive hits (*FDX1, LIAS, LIPT1, DLD, DLAT, PDHA1, PDHB*) and negative hits (*MTF1, GLS, CDKN2A*), were divided according to the molecular function of cuproptosis‐related genes [18]. Copper enrichment was found in both serum and tumor tissue from patients with a variety of tumors [19, 20, 21]. At present, the research of cuproptosis in liver cancer, lung cancer, triple negative breast cancer and other tumors has been gradually carried out [22, 23, 24, 25]. Prognostic models based on cuproptosis characteristics in various tumors are mostly constructed from one aspect, with few integrating clinical features, molecular landscapes, and genetic mutation features to construct models, and there is a lack of validation through molecular biology experiments [26, 27, 28, 29, 30]. Research in the field of liver cancer has made rapid progress, with studies constructing prognostic models and verifying that copper death‐related gene *LIPT1* may promote the proliferation, invasion, and migration ability of liver cancer cells [31]. Other studies have demonstrated that copper can mediate copper‐dependent cell death to suppress the proliferation of breast cancer cells and decrease tumor volume before breast cancer surgery through the development of a hydrogel system [32]. In the field of breast cancer, the latest research found that copper sensitization system therapy can target and induce copper poisoning programs in internal and external tumor cells, and significantly inhibit lung metastasis of breast cancer [33]. In gastric cancer, the construction of prognosis models related to copper mortality features is not complete, and there is a lack of prognosis models based on the clinical characteristics and detailed molecular landscape features of gastric cancer patients. However, it remains to be explored in the development of molecular‐targeted therapy for gastric cancer.

In this study, we delved into the interactions among cuproptosis‐related genes, considering them as a cuproptosis‐related signature to investigate their association with prognosis and molecular pathways in GC. A novel index termed as “cuproptosis signature risk score (CSRS),” which based on the principle components of the cuproptosis‐related transcriptomic, was utilized for evaluation of cuproptosis risk and survival conditions. We conducted an analysis of the genetic mutation landscape associated with cuproptosis in distinct risk subtypes of GC patients, revealing marked differences between the two subtypes. Our predictive model holds promise for improved prognosis prediction in patients, carrying significant clinical implications.

## RESULTS

### The landscape of the mutation of cuproptosis‐related genes in gastric cancer

**Figure 1:**
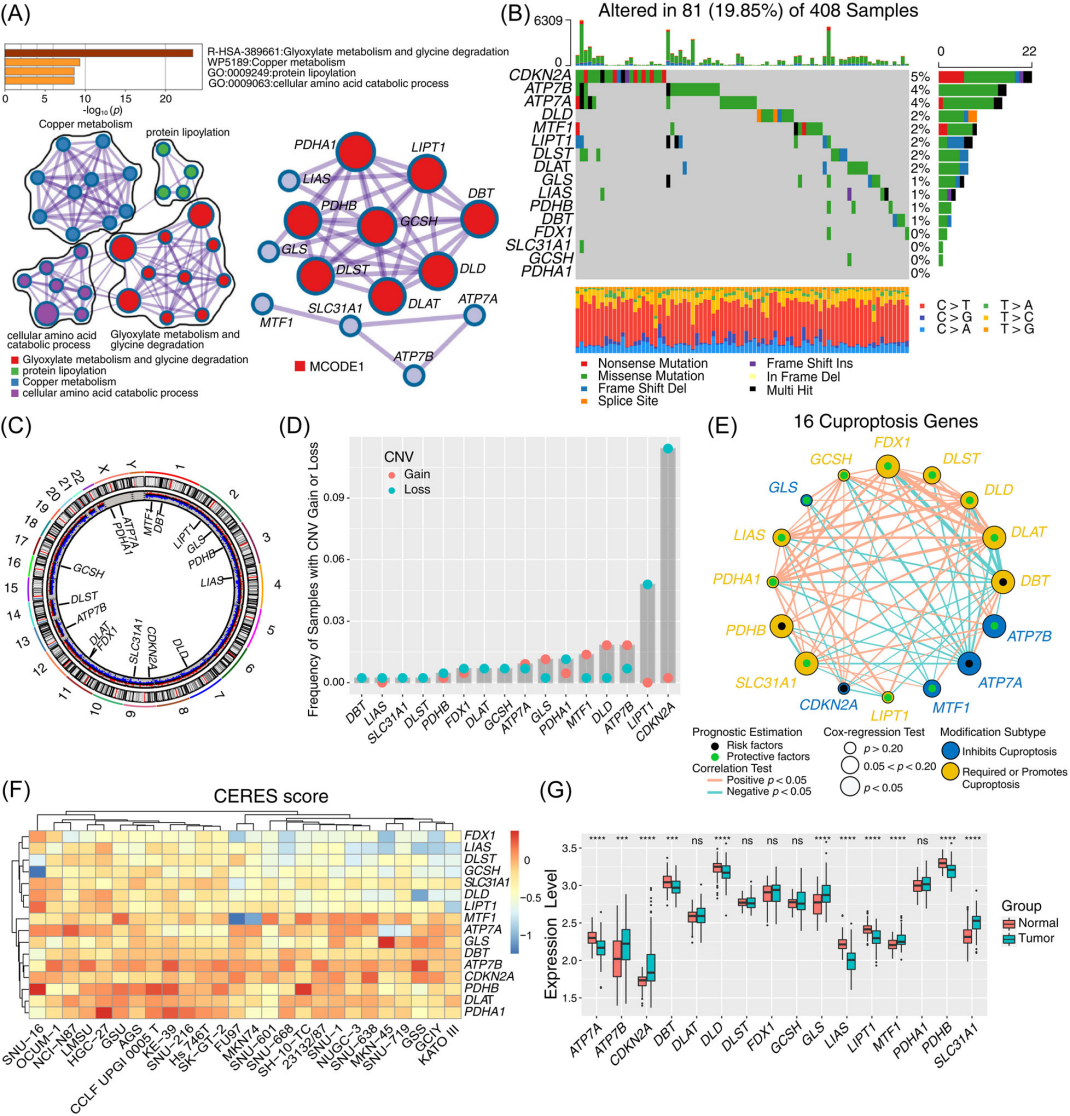
Comprehensive characteristics of cuproptosis‐related genes in gastric cancer. (A) Metascape enrichment network visualization showed that the intra‐cluster and intercluster similarities of enriched terms of 16 copper‐mediated cytotoxicity‐related genes. Different clusters were coded in different colors. (B) 81 of the 408 gastric cancer (GC) patients underwent genetic alterations on 16 cuproptosis‐related genes, with a frequency of 19.85%, mostly consisting of missense mutations, nonsense mutations, and insertion mutations. The number on the right panel indicated the mutation frequency of each cuproptosis‐related gene. The distribution below the chart shows the mutation frequency of the single base group. Each column represented single patients. (C) The location of copy number variation (CNV) alteration of cuproptosis‐related genes on chromosomes. (D) The CNV gain or loss of 16 cuproptosis‐related genes in GC. The loss of CNV was labeled with pink dot; the gain of CNV was labeled with blue dot. (E) The correlation and prognosis of 16 cuproptosis‐related genes in GC. The cuproptosis‐related genes in different functions were depicted by circles in different colors. The lines linking cuproptosis‐related genes represented their interaction with each other. The size of each circle represented the prognosis effect of each gene with scaled log‐*p* value. Protective factors for patients' survival were indicated by a green dot in the circle center and risk factors indicated by the black dot in the circle center. (F) The influence of knockout of 16 cuproptosis‐related genes on the proliferation in gastric cancer cell lines. The lower the CERES score, the greater the effect after the gene knockout. (G) The difference in mRNA expression levels of 16 cuproptosis‐related genes between paired normal and GC samples. The asterisks represented the statistical *p* value (*p < 0.05; **p < 0.01; ***p < 0.001).

In this study, we investigated the roles of 16 cuproptosis‐related genes in GC. GO enrichment and Metascape analyses were performed on 16 cuproptosis‐related genes, revealing significantly enriched biological processes that are summarized in Figure 1A. These genes were mainly enriched in glyoxylate metabolism, glycine degradation, copper metabolism, and so forth. Genetic alterations, predominantly missense mutations, insertional mutations, and frameshift mutations, were observed in 81 of 408 (19.85%) samples of cuproptosis‐related genes. The most common base mutations are C to T, T to C, and C to A (Figure 1B). Among the cuproptosis‐related genes, *CDKN2A* exhibited the highest mutation frequency, closely followed by *ATP7B* and *ATP7A*. We further investigated the co‐occurrence of mutations among all cuproptosis‐related genes and identified significant mutation co‐occurrence relationships between *CDKN2A* and *ATP7A, ATP7B* and *LIPT1, ATP7A* and *ATP7B*, as well as *ATP7A* and *LIPT1* (Figure S1A and Table S1). Upon further analysis of the 16 cuproptosis‐related genes, it was evident that CNV mutations were prevalent. Figure 1C illustrates the chromosomal locations of CNV alterations for these genes. Additionally, our analysis indicated that the frequencies of CNVs across the 16 cuproptosis‐related genes were inconsistent, suggesting variable rates of mutation across the gene set. *CDKN2A* and *LIPT1* had prevalent CNV deletions, whereas the *ATP7B, DLD, MTF1, GLS*, and *ATP7A* showed widespread CNV amplification (Figure 1D).

Furthermore, to assess the mutual regulation among the cuproptosis‐related genes, Spearman correlation analysis was conducted. The results revealed a significant negative correlation between *DBT* and *ATP7A* with other cuproptosis‐related genes. Conversely, *DLAT, FDX1*, and *PDHA1* demonstrated a significant positive correlation with other genes associated with cuproptosis (Figure S1B). Cox regression analysis was employed to determine the association between cuproptosis‐related genes and the prognosis of gastric cancer (GC) patients. The forest plot generated from this analysis indicated that *DBT, ATP7A, PDHB*, and *CDKN2A* could be regarded as risk factors significantly linked to overall survival in GC patients (Figure S1C). The interaction relationships between 16 cuproptosis‐related genes were also visualized in the form of a relationship network, whose lines representing the correlation between the two genes and circle size representing the prognosis value (Figure 1E). The intricate wiring highlighted 16 cuproptosis‐related genes acting together to influence the development and progression of gastric cancer and also provided a rationale for subtyping based on cuproptosis signature. Average gene essentiality scores, specifically CRISPR‐Cas9 gene knockout scores (CERES), were computed across 26 cell lines originating from stomach adenocarcinoma (STAD). These scores serve as a metric to assess the degree of gene dependence in these cell lines. *FDX1, LIAS, DLST, GCSH*, and *DLD* in the majority of cell lines were below −0.25 score and had a potential impact on cell proliferation (Figure 1F). The expression patterns of cuproptosis‐related genes differed notably between tumor specimens and adjacent normal specimens. Specifically, genes such as *LIPT1, ATP7A, LIAS, DBT*, and *PDHB* were found to be significantly downregulated in the tumor tissue. Conversely, genes like *CDKN2A, GLS, MTF1*, and *SLC31A1* exhibited marked upregulation in the tumor, suggesting distinct roles in the pathophysiology of the disease (Figure 1G).

### Cuproptosis signature patterns characterized by specific clinical features and molecular subtypes

**Figure 2:**
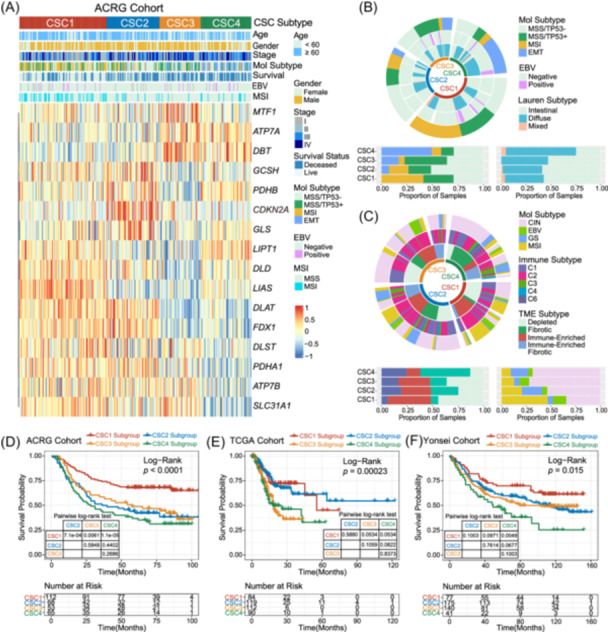
Cuproptosis signature patterns characterized by specific clinical features and molecular subtypes. (A) Heatmap shows the scaled expression of representative 16 cuproptosis‐related genes curated from the Asian Cancer Research Group (ACRG) in distinct cuproptosis signature clusters. The clinical features and subtypes, including age, gender, stage, molecular subtype, Epstein–Barr virus (EBV), and microsatellite instability (MSI) were annotated with different colors. (B, C) Comparison of cuproptosis signature clusters with different molecular subtypes of gastric cancer in ACRG (B) and The Cancer Genome Atlas (TCGA) (C) cohort. (D‐F) Kaplan–Meier curves of overall survival (OS) for 298 gastric cancer (GC) patients in ACRG cohort (D), 405 GC patients in TCGA cohort (E), and 433 GC patients in Yonsei cohort (F) with different cuproptosis signature clusters (log‐rank test).

The relationships between the expression patterns of cuproptosis‐related genes and clinical characteristics, as well as molecular subtypes, were further investigated in GC tumors. Based on the 16 cuproptosis‐related signature genes, we obtained four stable transcriptomic clusters by unsupervised clustering analysis (Figure S2A,B). We observed notable differences in the expression patterns of cuproptosis‐related genes across distinct clusters, indicating potential variations in the underlying biological processes and mechanisms associated with these genes in different subpopulations of gastric cancer. *LIAS, DLAT, PDHA1*, and *SLC31A1* were significantly elevated in the CSC1 subtype; *CDKN2A* and *GLS* were markedly increased in the CSC2 subtype; *MTF1, DBT*, and *SLC31A1* were evidently increased in the CSC3 subtype; *ATP7A* and *LIPT1* were obviously increased in the CSC4 subtype (Figure 2A, ACRG cohort). A similar expression distribution of the cuproptosis‐related genes was also observed in The Cancer Genome Atlas (TCGA) and Yonsei cohort (Figure S2C,D). Interestingly, a preponderance of microsatellite instability (MSI)‐positive tumors converged within the CSC1 subtype, culminating in the amalgamation of a hypermutated phenotype, MSI, and an immune‐enriched subtype. Conversely, the CSC4 subtype exhibited a strong correlation with advanced tumor staging, the mesenchymal phenotype, the diffuse histological subtype, and a fibrotic tumor microenvironment (TME) subtype. These observations underscore the intricate relationship between genetic instability, tumor microenvironment, and gastric cancer progression (Figure 2B,C), which indicated stromal invasion and worse prognosis. Further survival analysis uncovered marked prognostic disparities amongst the four clusters defined by cuproptosis signatures in gastric cancer (GC) samples. Notably, the CSC1 signature demonstrated a favorable prognostic correlation, whereas the CSC4 signature was associated with adverse survival outcomes (Figure 2D–F and Table S2). Multivariate Cox proportional hazards regression analysis further confirmed that this stratification model exhibited an independent association with patient survival outcomes (ACRG cohort: CSC1 vs. CSC4, hazards ratio [HR], 2.24 [95% CI, 1.43–3.50], *p* < 0.001, Figure S2E; TCGA cohort: CSC1 vs. CSC4, HR, 2.31 [95% CI, 1.32–4.02], *p* = 0.003, Figure S2F; Yonsei cohort: CSC1 vs. CSC4, HR, 1.85 [95% CI, 1.08–3.19], *p* = 0.026, Figure S2G;). Additionally, we examined the distribution in cuproptosis scores obtained through single sample gene set enrichment analysis (ssGSEA) across different clusters. Our analysis revealed significant differences in the distribution of cuproptosis scores among clusters in all three datasets. Specifically, the scores for CSC3 and CSC4 were found to be significantly lower compared to CSC1 and CSC2 (Figure S2H–J).

### Cuproptosis signature clusters characterized by distinct molecular processes and genomic alterations

**Figure 3:**
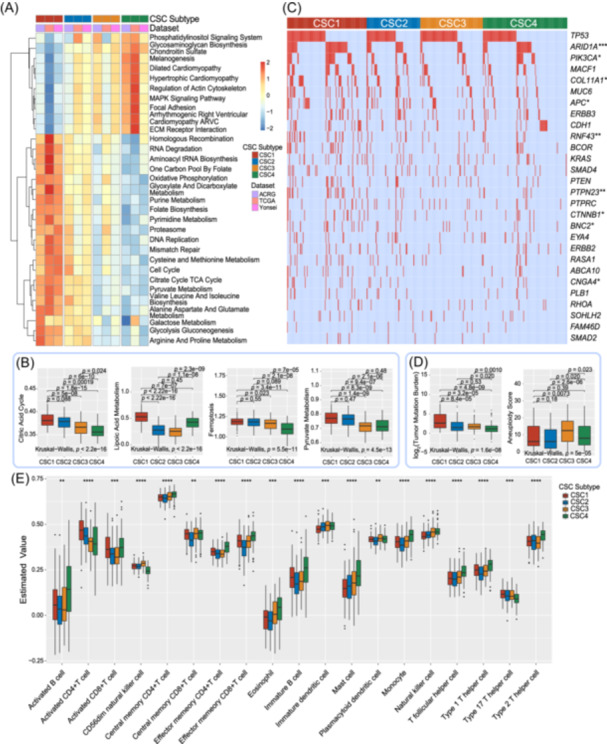
The cuproptosis signature clusters characterized by distinct functional signal enrichment and immune landscapes. (A) Heatmap shows the enrichment signal pathways of Kyoto Encyclopedia of Genes and Genomes (KEGG) in four different cuproptosis signature clusters across three datasets. (B) Distribution of molecular signatures curated from Zeng et al. study, including citric acid cycle, ferroptosis, lipoic acid metabolism and pyruvate metabolism among CSC1, CSC2, CSC3, and CSC4 subtypes in Asian Cancer Research Group (ACRG) cohort. (C) The mutational landscape of significantly mutated genes (SMGs) of The Cancer Genome Atlas (TCGA) gastric cancer in four cuproptosis signature clusters. This waterfall plot depicts the mutation frequency of SMGs across four clusters, and genes with statistically significant differential distribution are highlighted in upper right asterisk. (D) Tumor mutation load and aneuploidy score among CSC1, CSC2, CSC3, and CSC4 subtypes were compared in ACRG cohort. (E) Comparison of the fraction of single sample gene set enrichment analysis (ssGSEA) algorithm annotated cell subsets in four cuproptosis signature clusters. Within each group, the thick line represented the median value. The bottom and top of the boxes were the 25th and 75th percentiles (interquartile range). The whiskers encompassed 1.5 times the interquartile range. The statistical difference of four clusters was compared through the Kruskal–Wallis *H* test. *p < 0.05; **p < 0.01; ***p < 0.001.

To investigate the underlying biological molecular changes of the four clusters based on cuproptosis signatures, we conducted gene set variation analysis (GSVA) enrichment analysis using the Kyoto Encyclopedia of Genes and Genomes (KEGG) gene set. The results of GSVA revealed that CSC1 exhibited significant enrichment in processes related to substance synthesis, metabolism, and energy metabolism, including aminoacyl‐tRNA biosynthesis, one‐carbon pool by folate, glyoxylate and dicarboxylate metabolism, arginine and proline metabolism, citrate cycle (TCA cycle), and oxidative phosphorylation. Conversely, CSC4 predominantly showed enrichment in pathways associated with cell adhesion and stromal pathways, such as glycosaminoglycan biosynthesis (chondroitin sulfate), regulation of actin cytoskeleton, focal adhesion, and ECM‐receptor interaction. These findings suggest distinct biological characteristics and potential functional roles of different clusters in gastric cancer. Interestingly, all of these relevant biological processes were median enriched in CSC2 subtype (Figures 3A and S3A). Moreover, we utilized the immuno‐oncology signatures, which curated from Zeng et al. study [34], to evaluate the biological process and pathway across four CSC subtypes. CSC1 and CSC2 had a higher citric acid cycle score in the 3 databases (Figures 3B and S3B,F); CSC1 had a higher lipoic acid metabolism in the 3 databases (Figures 3B and S3C,G); CSC4 had a lower ferroptosis score in ACRG cohort (Figure 3B), and CSC1 had a higher ferroptosis score in TCGA cohort (Figure S3E); CSC1 and CSC2 had a higher pyruvate metabolism score (Figures 3B and S3D,H); Glyoxylate and dicarboxylate metabolism score was decreased progressively from CSC1 to CSC4 in Yonsei cohort (Figure S3I). To gain deeper insights into the mutational landscape across distinct CSC subtypes, we conducted SMG analysis within the TCGA cohort, comparing gene mutation frequencies among CSC1, CSC2, CSC3, and CSC4 subsets. Our analysis revealed that in the CSC1 subtype, *ARID1A, PIK3CA, COL11A1*, and *APC* exhibited higher mutation rates compared to the other subtypes within the TCGA cohort. These findings suggest potential subtype‐specific mutational patterns that may contribute to the unique characteristics and behaviors of CSC1 in gastric cancer (adjusted chi‐square test, *p* < 0.05; Figure 3C).

We also explored the tumor microenvironment alterations in different CSC subtypes. Genomic analysis revealed that CSC1 exhibited the highest mutational load and CSC3 had a higher aneuploidy score (Figure 3D). Antitumor lymphocyte cell subpopulations, such as activated CD4^+^/CD8^+^ T cells were mainly enriched in the CSC1 subtypes. However, effector memory CD4^+^/CD8^+^ T cells, mast cells, eosinophils, Type 1 helper cells, and Type 2 T helper cells were markedly elevated in the CSC4 subtype (Figure 3E).

### Construction of cuproptosis signature risk score and exploration of its clinical relevance

**Figure 4:**
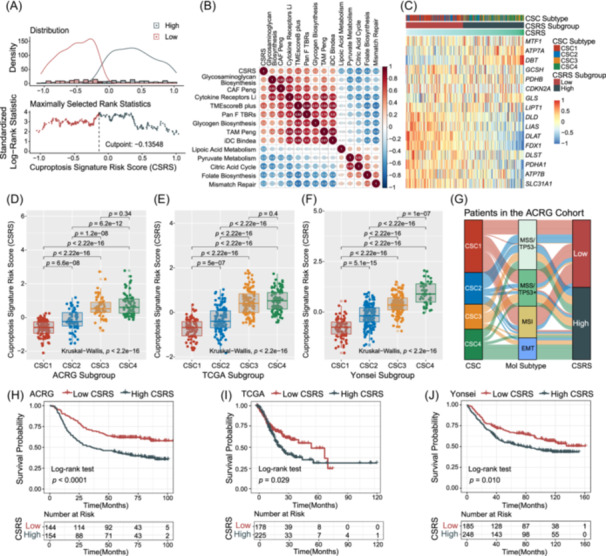
Construction of the cuproptosis signature risk score and exploration of its clinical relevance. (A) The cutoff point of the cuproptosis signature risk score identified by using the standardized maximally selected log‐rank statistics. (B) Correlations between cuproptosis signature risk score (CSRS) score and the known biological gene signatures using Spearman analysis. The negative correlation was marked with blue and positive correlation with red. Number located in the circle indicated the correlation coefficients. (C) Heatmap shows the scaled expression level of cuproptosis‐related genes and CSRC score among four CSC subtypes and two CSRS subtypes. Each column represented single patients. (D–F) Distribution of cuproptosis signature risk scores among four cuproptosis signature clusters in three independent datasets of Asian Cancer Research Group (ACRG) (D), The Cancer Genome Atlas (TCGA) (E), and Yonsei (F). (G) Alluvial diagram of cuproptosis clusters in groups with different molecular subtypes (MSS/TP53−, MSS/TP53+, microsatellite instability [MSI], epithelial–mesenchymal transition [EMT]), cuproptosis signature clusters (CSC1–CSC4), and cuproptosis signature risk scores subtype (Low and High). (H–J) Kaplan–Meier curves for high and low CSRS patient groups in the three independent datasets of ACRG (H), TCGA (I), and Yonsei (J). Log‐rank test, *p* < 0.05.

Despite our findings highlighting the prognostic significance of cuproptosis signature‐based clusters, these analyses were limited to the patient population level and lacked precision in predicting cuproptosis signature patterns within individual tumors. To address this limitation, we devised a scoring system, termed the cuproptosis signature risk score (detailed in the Method section), aimed at quantifying the cuproptosis signature in individual GC patients. This scoring scheme enables a more personalized assessment of cuproptosis‐related risk, facilitating tailored therapeutic approaches in gastric cancer management. In principal component analysis and the 16 cuproptosis‐related genes contributing to the top 5 principal components, the top‐ranked genes include *DLAT, FDX1, DBT, DLD*, and so forth (Figure S4A). We further divided the GC patients into CSRS‐Low and CSRS‐High subtypes. The cutoff point was identified by using standardized maximally selected log‐rank statistics in ACRG cohort (Figure 4A). We examined the relationship between known biological signatures and the cuproptosis signature risk score through Spearman analysis. The resulting heatmap of the correlation matrix revealed notable correlations. Specifically, the cuproptosis signature risk score was markedly negatively correlated with lipoic acid, pyruvate metabolism, and the citric acid cycle. Conversely, it showed a positive correlation with signatures related to cancer fibroblasts, tumor‐associated macrophages, glycogen and glycosaminoglycan biosynthesis, as well as cytokine receptors. These findings suggest complex interactions between the cuproptosis‐related risk and various biological processes in gastric cancer, pointing to potential targets for further investigation and therapeutic intervention (Figure 4B and Table S3). Our observations indicate a noteworthy difference in the expression levels of cuproptosis‐related genes between the CSRS‐Low and CSRS‐High subtypes. *DBT, MTF1*, and *ATP7A* were significantly elevated in the CSRS‐High subtype; *ATP7B, SLC31A1, GCSH, LIAS, DLAT, FDX1, DLD*, and *PDHA1* were obviously increased in the CSRS‐Low subtype in the 3 databases (Figures 4C and S4B,C). We also explored the distribution between the cuproptosis signature risk scores and CSC clusters and found that the cuproptosis signature risk score was significantly increased in CSC4 subtypes than the other 3 subtypes among the three databases (Figure 4D–F). Considering the intricacies involved in quantifying the cuproptosis signature, we have employed an alluvial diagram to illustrate the workflow of constructing the cuproptosis signature risk score (Figure 4G). By presenting this workflow in a clear and concise manner, the alluvial diagram aids in understanding the methodology behind the cuproptosis signature risk score and facilitates the replication of our approach in future studies. To further validate the prognostic value of the cuproptosis signature risk score, we stratified patients into two subtypes: CSRS‐Low and CSRS‐High. As hypothesized, patients classified as CSRS‐Low demonstrated a significantly better prognosis across all three cohorts studied. This finding underscores the potential of the cuproptosis signature risk score as a predictor of survival outcome in gastric cancer patients (Figure 4H–J and Table S2). Multivariate analysis of the three cohorts has indeed confirmed that the cuproptosis signature risk scores can serve as an independent prognostic biomarker in GC (HR, 1.61 [95% CI, 1.16–2.25], *p* = 0.005, ACRG cohort in Figure S4D; HR, 1.73 [95% CI, 1.19–2.50], *p* = 0.004, TCGA cohort in Figure S4E; HR, 1.49 [95% CI, 1.12–2.00], *p* = 0.007, Yonsei cohort in Figure S4F).

### Tumor genomic landscapes in cuproptosis signature mutation gastric cancer

**Figure 5:**
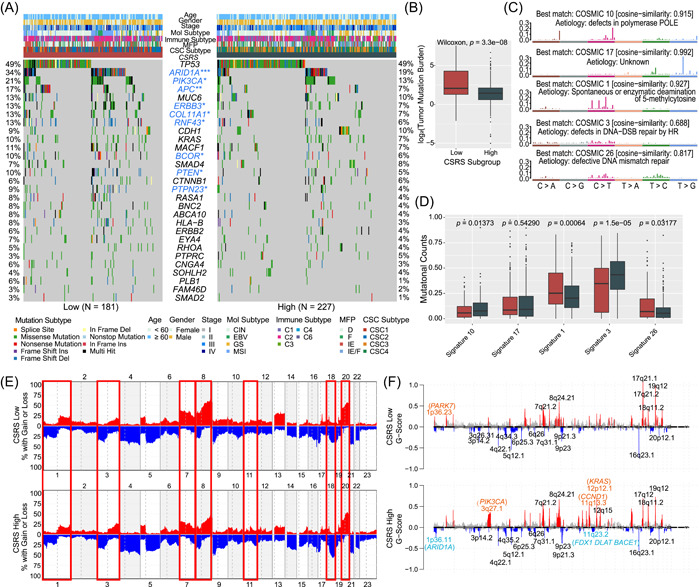
Tumor genomic landscapes in different cuproptosis signature risk score (CSRS) subtype of gastric cancer. (A) The mutational landscape of significantly mutated genes (SMGs) in The Cancer Genome Atlas (TCGA)‐stomach adenocarcinoma (STAD) stratified by low (left panel) versus high CSRS (right panel) subtypes. Individual patients were represented in each column. Mutational frequencies of SMGs in different CSRS subtypes were depicted in two sides of the panel. Genes were highlighted in blue for those statistically significant by Fisher's exact test. *p < 0.05; **p < 0.01; ***p < 0.001.  Age, gender, stage, molecular subtype, immune subtype, MFP and CSC were shown as patient annotations in the upper panel. (B) Comparison of tumor mutation load in CSRS‐High versus low subtypes. (C) The mutational activities of the corresponding extracted mutational signatures (Catalog of Somatic Mutations in Cancer [COSMIC] 10, 17, 1, 3, and 26, named as COSMIC database). (D) Comparison of mutational activities of corresponding extracted mutational signatures in high versus low CSRS subtypes. (E) Arm‐level somatic copy‐number alteration (SCNA) events in high versus low CSRS subtypes. Red denotes amplification and blue denotes deletion. (F) Focal peaks with significant somatic copy‐number amplification (red) and deletions (blue) (*Q* values < 0.1) are shown. The top 20 amplified and deleted cytobands are labeled. Representative genes encoded from these focal peaks are highlighted in approximate positions across the genome.

To gain deeper understanding of the genomic alterations associated with the cuproptosis signature risk score in gastric cancer patients, we analyzed somatic mutation data obtained from whole‐exome sequencing (WES‐seq) and single‐nucleotide polymorphism (SNP) array analysis. Specifically, we conducted a significantly mutated gene (SMG) analysis in the TCGA cohort, comparing the frequency of gene mutations between the CSRS‐Low and CSRS‐High subtypes. This comprehensive approach allowed us to identify genetic aberrations that may underlie different risk scores and potentially contribute to the pathogenesis and progression of gastric cancer. The mutational profile in the TCGA cohorts showed that *ARID1A, PIK3CA, APC, ERBB3, COL11A1, RNF43, BCOR, PTEN*, and *PTPN23* had higher mutation rates in the CSRS‐Low subtype than in the CSRS‐High subtype (adjusted chi‐square test, *p* < 0.05, Figure 5A). A consistent result was also observed in the TCGA cohort, lower cuproptosis signature risk scores in patients were significantly associated with higher tumor mutational load (Figure 5B). After parsing the somatic mutation data from whole‐exome sequencing (WES‐seq) and single‐nucleotide polymorphism (SNP) array analysis, we proceeded to analyze the single‐nucleotide variants (SNVs) in gastric cancer (GC) tumors. Specifically, we compared the SNV profiles of CSRS‐Low and CSRS‐High subtypes across a matrix of 96 possible mutations. The pie chart shows that the CSRS‐Low subtype had a slight decrease in C > T transitions and the CSRS‐Low subtype had a slight increase in T > G transitions. The Lego plot analysis reveals distinct mutational patterns in gastric cancer (GC), with C > T transitions predominantly occurring at ApCpN trinucleotide sites. Notably, a specific mutation, T > G transition at GpTpG sites, is highlighted in the CSRS‐Low subtype (Figure S5A). Subsequently, we extracted five mutational signatures from the genomic data (Figures S5B and 5C), including defects in polymers POLE (COSMIC 10), spontaneous or enzymatic deamination of 5‐methylcytosine (COSMIC 1), defects in the DNA‐DSB repair by HR (COSMIC 3), defective DNA mismatch repair (COSMIC 26) and an unknown one (COSMIC 17) (Figure 5C). We found that CSRS‐High subtype had higher mutational counts in signature 1 and signature 26; however, lower mutational counts in signature 3 and signature 10 (*p* < 0.05, Figure 5D). The arm‐level somatic copy number alteration (SCNA) results suggest that specific chromosomal regions, including chr1p, chr3, chr7q, chr8q, chr11q, chr18q, and chr20p, contain the most frequently amplified or deleted regions in GC. These findings are significant because they implicate these chromosomal regions in the pathogenesis and progression of GC. Amplifications and deletions in these regions may lead to the dysregulation of oncogenes or tumor suppressor genes, respectively, driving tumor growth and metastasis (Figure 5E). The top‐ranked CNV sites in two CSRS subtypes were 8q, 9p, 20q, and so forth (Figure S5D). The focal level SCNAs revealed that the cytobands in *1p36.23* (*PARK7*) in CSRS‐Low subtype, and *3q27.1* (*PIK3CA*), *11q13.3* (*CCND1*), and *12p12.1* (*KRAS*) in cuproptosis signature risk high score subtype contained the markedly amplified focal regions; and cytobands in *1p36.11* (*ARID1A*) and *11q23.2* (*FDX1, DLAT, BACE1*) in cuproptosis signature risk high score subtype contained the frequently deleted regions (FDR < 0.05, Figure 5F). Mutation counts attributed to the COSMIC 1 and COSMIC 26 signatures exhibited a notable elevation in the CSRS‐Low subtype, while a conspicuous reduction in mutation counts linked to the COSMIC 3 signature was observed in the CSRS‐High subtype (Figure S5C).

### Analysis of correlation and effectiveness between CSRS score and antineoplastic drugs

**Figure 6:**
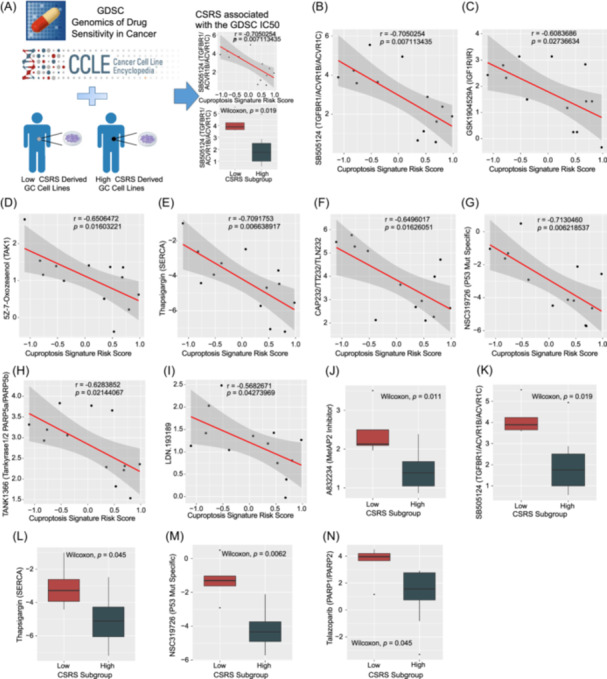
Exploration of potential agents in the treatment of gastric cancer (GC) patients with high cuproptosis signature risk score (CSRS) scores. (A) Schematic of drug association and sensitivity analyses in relation to CSRS score in GC cell lines. (B–I) The correlation analysis between CSRS and transforming growth factor‐β (TGF‐β) receptor antagonists, GSK1904529A(IGF1R/IR), 5Z.7. Oxozeanol (TAK1), thapsigargin (SERCA), CAP232/TT232/TLN232, NSC319726 (P53 Mutant Specific) and TANK1366 (Tankyrase1/2 PARP5a/PARP5b) in GC. (J–N) Comparison of drug effectiveness (A832234 [MetAP2 Inhibitor], SB505124 [TGFBR1/ACVR1B/ACVR1C], thapsigargin [SERCA], NSC319726 [P53 Mutant Specific] and talazoparib [PARP1/PARP2]) between low and high subtypes of CSRS in GC.

We further jointly analyzed the Cancer Cell Line Encyclopedia (CCLE) and Genomics of Drug Sensitivity in Cancer (GDSC1) databases to determine the association between CSRS score and antineoplastic drug sensitivity of gastric cancer cell lines (Figure 6A and Table S4). Considering the worse prognosis, we concentrated particularly on identifying the potential drug regimens for GC samples with high CSRS scores. Meaningfully, there was a significant negative correlation between the CSRS score and the IC50s of select drugs, such as SB505124 (transforming growth factor‐β [TGF‐β] receptor antagonists, Figure 6B, Spearman correlation test), GSK1904529A (IGF1R/IR inhibitor, Figure 6C), 5Z‐7‐Oxozeaenol (TAK1 inhibitor, Figure 6D), thapsigargin (SERCA inhibitor, Figure 6E), CAP232/TT232/TLN232 (Glycolysis, SSTR1/SSTR4 activator, Figure 6F), NSC319726 (P53‐R175 mutant activator, Figure 6G), TANK1366 (Tankyrase1/2, PARP5a/PARP5b inhibitor, Figure 6H), and LDN193189 (BMP ALK2/ALK3 inhibitor, Figure 6I). Further analysis showed that these agents have a lower IC50 value in CSRS‐High subtype patients, such as A832234 (MetAP2 inhibitor, Figure 6J, Wilcoxon test), SB505124 (Figure 6K), thapsigargin (Figure 6L), NSC319726 (Figure 6M), and talazoparib (PARP1/PARP2 inhibitor, Figure 6N). Interestingly, SB505124, thapsigargin, and NSC319726 appeared in both analyses.

### Verifying the differences between CSRS‐Low (AGS) and CSRS‐High (HGC‐27) subtypes through Western blot experiments and drug sensitivity experiments

**Figure 7:**
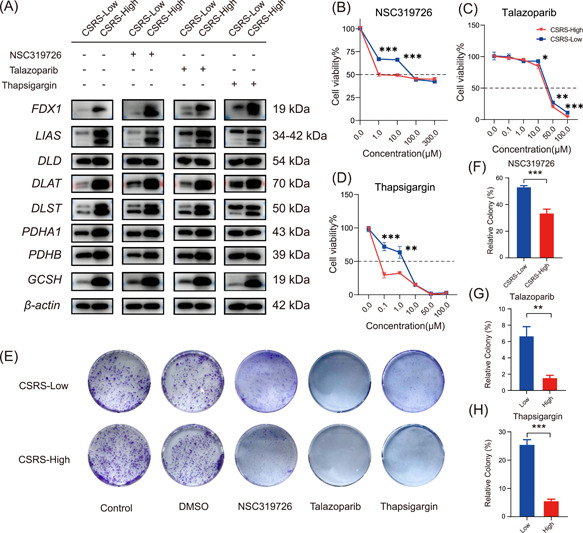
Differences in expression and drug sensitivity of cuproptosis‐related proteins in gastric cancer cells between the cuproptosis signature risk score (CSRS)‐Low (AGS) and CSRS‐High (HGC‐27) subtypes. (A) Western blot analysis of FDX1, LIAS, DLAT, DLST, GCSH, DLD, PDHB, PDHA1, and β‐actin in the CSRS‐Low (AGS) and CSRS‐High (HGC‐27) subtypes. (B–D) Sensitivity analysis of NSC319726, talazoparib and thapsigargin drugs in gastric cancer cells of the CSRS‐Low (AGS) and CSRS‐High (HGC‐27) subtypes. (E–H) Plate colony of two gastric cancer (GC) cell lines of the CSRS‐Low (AGS) and CSRS‐High (HGC‐27) subtypes treated with dimethyl sulfoxide (DMSO) or three drugs as indicated. *p < 0.05; **p < 0.01; ***p < 0.001.

We conducted molecular experiments to validate our findings, and the results of Western blot analysis revealed notable disparities in the expression levels of FDX1, LIAS, DLAT, DLST, GCSH, PDHB and PDHA1 in gastric cancer cells between the CSRS‐Low (AGS) and CSRS‐High (HGC‐27) subtypes. In the CSRS‐High subtype of gastric cancer cells, the expression of proteins related to cuproptosis progression was significantly increased, while the CSRS‐Low subtype was opposite (Figure 7A). In the CSRS‐Low and CSRS‐High subtypes, the expression of copper death‐related proteins showed significant changes in some molecules after drug stimulation, and the specific mechanism needs further exploration. All three drugs can effectively inhibit the proliferation of two GC cell lines tested, and all three drugs have good inhibitory effects. Additionally, NSC319726, talazoparib, and thapsigargin have relatively high IC50s in the CSRS‐Low subtype of gastric cancer cells (Figure 7B–D). The inhibitory effect of the drugs on the growth of gastric cancer cells was further confirmed by colony–forming assays (Figure 7E–H).

## DISCUSSION

An increasing number of studies have shown that the cellular cuproptosis mechanism plays a great role in tumor development and progression [35]. The utilization of multi‐omics technology has enabled the identification of cancer biomarkers generated by tumor cells within the intricate tumor microenvironment, thereby significantly advancing the development of novel diagnostic and therapeutic approaches [36]. There have been studies constructing prognosis models based on copper death features, but they have not comprehensively considered clinical features, and the explanation of the genetic molecular landscape is not in‐depth enough [37]. There is no cross‐validation between drug screening and cell line sensitivity, and no sensitivity‐related experiments have been conducted to verify the screened drugs [38]. A column chart model combining risk scores and other clinical pathological features has been constructed based on copper death features and tumor microenvironment single cell profiles, but its application and screening of effective drugs based on clinical features and molecular landscapes of gastric cancer are not detailed enough [39]. In contrast to this study, which solely utilized the TCGA data set for analysis, our study takes a more comprehensive approach by incorporating three different datasets. Additionally, our study employs a different modeling approach (PC), which may provide unique insights and perspectives on the topic at hand [40]. Part of these studies have explored the relationship between cuproptosis mechanisms and tumor genomics, but have not characterized the genomic landscape of specific tumors exhaustively. By considering multiple datasets and adopting a different methodology, our study aims to provide a more comprehensive and nuanced understanding of the subject matter. In this paper, we show that the cuproptosis signature is significantly associated with molecular landscapes and clinical characteristics of gastric cancer, which will strengthen our understanding of occurrence and development of copper‐mediated cytotoxicity and treatment schedule of gastric cancer.

In this study, utilizing an integrative clustering algorithm with cuproptosis‐related genes, we delineated four distinct cuproptosis signature subtypes. Notably, among these genes, *GLS* encodes glutaminase, a pivotal enzyme catalyzing the conversion of glutamine to glutamate. Glutamine metabolism is a crucial process in cancer cell proliferation and viability, highlighting the potential significance of *GLS* in this context [41]. *FDX1* is a gene that encodes the protein ferredoxin 1, which is involved in electron transfer reactions in the mitochondria. Abnormal *FDX1* expression has been associated with colorectal cancer and can affect cellular energy metabolism and redox balance [42]. *SLC31A1* is a gene that encodes the copper transporter protein *CTR1*. *ATP7B*, and *ATP7A* are genes that encode copper‐transporting ATPases involved in copper homeostasis. Dysregulation of *SLC31A1, ATP7B*, and *ATP7A* has been implicated in colorectal cancer and can affect copper homeostasis and tumor growth [43, 44]. *MTF1* is a gene that encodes the metal‐responsive transcription factor 1, which plays a role in regulating the expression of genes involved in metal ion homeostasis and oxidative stress response [45]. It can affect cellular response to oxidative stress and DNA damage. *DBT, LIPT1, CDKN2A, PDHB, DLD, DLAT, LIAS*, and *DLST* genes are involved in various cellular processes, including energy metabolism, DNA repair, and cell cycle regulation [35]. They have been implicated in colorectal cancer and can contribute to tumor growth and progression. These 16 cup‐associated genes differ in characteristics and mechanisms. The four subtypes had different molecular characteristics, immunological phenotypes, biological features, genomic features, and clinical prognosis in GC. CSC1 is mainly characterized by MSI molecular status, which corresponds to the immune enrichment phenotype. It is mainly enriched in the biological functions related to TCA cycle, pyruvate, and galactose metabolism et al. CSC4 is mainly characterized by epithelial–mesenchymal transition (EMT) molecular typing of gastric cancer, corresponding to the fibrous matrix phenotype and enriched in adhesion‐related biological functions. CSC2 and CSC3 were featured by intermediate conditions from CSC1 to CSC4. Previous studies have shown that the classification of tumor microenvironments can help us identify patients who may benefit from combined target molecular therapy [46, 47]. Based on these results, we speculated that GC patients with the CSC4 pattern may benefit from combination treatment with the blockade targets on adhesion‐related signaling pathways, and CSC1 patterns may benefit from blockade targets on tumor metabolism signaling. The results of immune infiltration showed a higher degree of T‐cell infiltration in CSC1 and a higher degree of B‐cell infiltration in CSC4. T cells are important for immune responses against pathogens and tumors. However, in colorectal cancer, their function can be suppressed, allowing the tumor to evade the immune system. Enhancing T cell function could improve colorectal cancer therapies. B cells, responsible for antibody responses, have a minor role in colorectal cancer. The role of B cells is relatively minor as this type of cancer typically does not trigger a strong antibody response. However, B cells may still contribute to the tumor microenvironment by modulating the immune response and promoting tumor growth [48]. The mechanism was consistent with the prognosis of CSC1 and CSC4. By exploring the differences in the immune microenvironment of different subtypes of tumors, we can provide theoretical support for immune‐targeted therapy for gastric cancer.

We further established a copper signature risk scoring system to evaluate patient prognosis and biological pathways and thus guide patient precise treatment. Further analysis revealed that patients with a higher CSRS score were mainly derived from CSC3 and CSC4 subtypes and had a worse prognosis. In the ACRG and Yonsei queues, the differences between CSC1 and CSC2, CSC3, and CSC4 are relatively significant. In the TCGA queue, there was no significant difference between the CSC1 and CSC2 groups, but a more significant difference compared to CSC3 and CSC4. The reason for this result may be that the TCGA cohort is mainly composed of Western populations, while the ACRG and Yonsei cohorts are mainly composed of Asian populations. Genomic analysis indicated that high CSRS subtype was characterized by decreased TMB and defective DNA mismatch repair signature. CNV with amplification of oncogenes, such as *PIK3CA, KRAS*, and *CCND1*, was found mainly focused in the CSRS‐High subtype; whereas the cuproptosis inhibitors, such as *FDX1* and *DLAT*, were markedly deleted in CSRS‐High subtype. These evidence suggest that the CSRS was associated with genomic alteration, and higher score indicated the increasing genetic heterogeneity. CRC patients who have a high TMB may exhibit increased tumor immunogenicity, making it more likely for tumor cells to be recognized and attacked by the immune system. Consequently, CRC patients with high TMB may display heightened sensitivity to immunotherapy and are more likely to benefit from immune checkpoint inhibitor therapy [49]. Mutations in the *PIK3CA* and *KRAS* genes are commonly found in colorectal cancer and are associated with increased tumor risk. *PIK3CA* mutations activate the PI3K signaling pathway, promoting tumor cell growth and survival. *KRAS* mutations lead to sustained activation of the KRAS protein, enhancing tumor cell growth and spread. Both mutations are associated with tumor aggressiveness, recurrence, and drug resistance in colorectal cancer [50]. Additionally, several chemical regimes mentioned above were found to be sensitive to high CSRS scores, indicating their potential significance in therapy.

Notably, this study found that certain cuproptosis‐related genes play important regulatory roles in the biological processes of tumors. Recent studies have found that the higher expression of copper transporter *ATP7A* can promote tumorigenesis and metastasis, and is significantly related to the poor prognosis of breast cancer patients [51]. Meanwhile, *ATP7A* can limit autophagy‐mediated degradation of *VEGFR2* and promote VEGFR signaling pathway and neoangiogenesis [52]. This study showed similar results, in which higher *ATP7A* enrichment was observed in the CSRS‐High GC samples. Previous studies have shown that *DLAT* was expressed in different gastric cancer types in different amounts, and found that knockdown of *DLAT* in *DLAT* high expression gastric cancer cell lines can affect the proliferative function of cells and may promote aerobic glycolysis pathway, oxidative phosphorylation and catabolic reactions [53]. PM2.5 exposure augmented the expression of the glycolytic gene *DLAT*, thereby stimulating the glycolytic process and ultimately facilitating the development of NSCLC [54]. Our results also showed that *DLAT* was mainly enriched in oxidative phosphorylation, citric acid cycle, pyruvate metabolism and other substances and energy metabolic processes in gastric cancer. *DLAT*‐related mechanisms are relatively complex and need to continue to be explored. Some studies have found that *FDXR* with *FDX2* deletion regulates *P73* tumor suppressor via *IRP2* to modulate aging and tumor suppression [55]. *FDXR* exerts its tumor suppressive effect by interacting with *P53* [56]. *FDX1* is closely related to glucose metabolism, amino acid metabolism and fatty acid oxidation and promotes ATP production in lung cancer, but it does not affect lung cancer cell proliferation and has no effect on apoptosis and cycle [57]. *FDX1* exhibits reduced expression in various cancer types and exhibits significant correlations with clinical parameters, TMB, MSI, and immune‐associated pathways [58]. Our study reveals that FDX1 is intimately linked to energy and substrate metabolism in gastric cancer, predominantly observed in patients with the CSRS‐Low subtype and associated with a more favorable prognosis. Collectively, these findings implicate that a holistic evaluation of cuproptosis attributes could bolster our comprehension of the underlying physiological mechanisms involved in cuproptosis.

Interestingly, our correlation and sensitivity analyses for antineoplastic drugs revealed several drugs with both correlation and sensitivity the same trend, such as SB505124, thapsigargin, and NSC319726. SB505124 is a selective TGFβR inhibitors acting on ALK4 and ALK5, which can induce cell death and inhibit biological functions such as tumor cell invasion, proliferation, and survival through a variety of mechanisms [59, 60]. Thapsigargin induces apoptosis in almost all cells, but recent studies have shown that it can inhibit Notch1 signaling pathway, and its benefits need to be comprehensively evaluated [59, 61]. NSC319726 can bind copper to induce oxidative stress leading to cell cycle arrest [62]. This can help us screen drug treatment targets for gastric cancer, and provide a theoretical basis for the selection of clinical precise treatment. And we preliminarily verified that the above three drugs have obvious effects on cell viability through molecular biology experiments, and then we need to verify the effects of the three drugs on cell proliferation, migration and invasion, cell cycle, apoptosis and other functions through experiments. Obviously, we need more evidence to support the clinical benefits of drugs.

## CONCLUSION

In this study, we reviewed the literature and collated 16 cuproptosis‐related gene signatures and identified that the cuproptosis stratification system was associated with different prognosis and molecular patterns. Although the results showed that model reliability and applicability were satisfactory, prospective clinical cohort studies are needed to validate the robustness of our model for clinical theragnostic, and subsequent studies focusing on biological experiments to validate its molecular mechanism in gastric cancer were needed to optimize the CSRS score system. Quantitative assessment of the CSRS in individual tumors will enhance our comprehension of cuproptosis occurrence, development, and treatment progression in GC. Encouragingly, cuproptosis‐based translational medicine exhibits potential as a clinical candidate, pending rigorous safety and efficacy evaluations in human cancer trials.

## METHODS

### Public data acquisition and data cleaning

We conducted a literature review focused on cuproptosis and compiled a list of 16 recognized cuproptosis‐related genes. These genes were then carefully analyzed to identify distinct clusters associated with the cuproptosis signature. The 16 cuproptosis‐related genes were divided into two groups, positive hits (*FDX1, DLST, LIAS, LIPT1, DLD, DLAT, DBT, SLC31A1, PDHB, PDHA1, GCSH*) and negative hits (*MTF1, GLS, CDKN2A, ATP7A, ATP7B*) (Table S5). Gastric cancer sample data with available transcriptomic and clinical data were obtained from public databases, including the ACRG data set (Asian Cancer Research Group, 2015, *N* = 298, https://www.ncbi.nlm.nih.gov/geo/query/acc.cgi?acc=GSE62254), TCGA‐STAD data set (The Cancer Genome Atlas‐Stomach Adenocarcinoma, 2018, *N* = 408, https://portal.gdc.cancer.gov/) and Yonsei data set (Yonsei University cohort, 2018, *N* = 433, https://www.ncbi.nlm.nih.gov/geo/query/acc.cgi?acc=GSE84437). A total of 1139 gastric cancer patients were included in the study for follow‐up analysis after removing the samples with incomplete information. The ACRG data set was employed for model establishment, and TCGA and Yonsei were used for further validation.

### Functional enrichment, interaction network analysis and pathway annotation

The Metascape database (https://metascape.org) was used for functional enrichment analysis of cuproptosis‐related genes. The protein–protein interaction network of 16 cuproptosis‐related genes was using the STRING (https://string-db.org) to plot, which was based on gene expression levels. The enrichment pathway of the selected genes was based on GO enrichment analysis.

### Copy number variation (CNV) analysis, high frequency mutant genes and tumor mutation characteristics

The R package ”oncoplot” was used to perform the gene mutation landscape descriptions. Mapping of 16 cuproptosis‐related genes to chromosomes was performed with the R package “RCircos.” CNV analysis was performed with the R package “maftools” to annotate amplifications and deletions cytobands in different CSRS subtype. The somatic copy number alterations (SCNA) and aneuploidy scores for each gastric cancer sample were compiled from previous research studies [63]. The Signature Enrichment function of Bayesian Inference for Nonnegative Matrix Factor Deconvolution Models was used to determine the optimal number of mutational signatures that could be extracted. Cosine similarity analysis was utilized in the Catalogue of Somatic Mutations in Cancer to compare and annotate the explored mutational signatures of gastric cancer.

### Unsupervised cluster analysis of 16 cuproptosis‐related genes

The iClusterPlus package implements a regularized latent variable model for clustering that incorporates joint inference across different data types to produce an integrated cluster assignment. This approach leverages information from multiple sources to improve the accuracy and robustness of clustering results. Here, we employed the iClusterPlus package to identify different clusters of cuproptosis signatures based on transcriptomic profile of 16 cuproptosis‐related genes in GC. According to the iClusterPlus guidelines, the optimal number of clusters was determined to be four, as indicated by the leveling off of the curve representing the percentage of explained variation. This suggests that further increasing the number of clusters beyond four would not significantly improve the amount of variation explained by the model. We utilized the iCluster2 function with *K* = 4, lambda set to 0.1, and method set to “lasso” to perform the clustering analysis.

### Establishment of the cuproptosis score via ssGSEA

We utilized a gene set comprised of cuproptosis genes and applied the ssGSEA method to assign a cuproptosis score to each sample. ssGSEA is a gene set‐based approach that evaluates the enrichment of a gene set within an individual sample. It quantifies gene set enrichment by calculating the rank order of genes within each gene set, resulting in a gene set enrichment score [64]. The key advantage of ssGSEA is its ability to assess gene set enrichment within a single sample, eliminating the need for inter‐sample comparisons. This is particularly valuable when working with a limited number of samples or lacking a control group. ssGSEA can be applied to diverse types of gene expression data, including RNA‐seq and microarray data.

### Immune cell infiltration estimation with ssGSEA

The relative infiltration of 28 immune cell types in the GC tumor microenvironment were quantified by the ssGSEA. Special feature gene panels for each immune cell subset were curated from Charoentong et al. research [65]. The relative abundance of each immune cell type was represented by an enrichment score in the ssGSEA analysis. The bio‐similarity of the immune cell filtration was estimated by multidimensional scaling (MDS) and a Gaussian fitting model.

### Cell lines and drug sensitivity analysis

We also evaluated the genetic vulnerabilities of the cuproptosis‐related proteins in the GC using data from the Cancer Dependency Map Project (DepMap). Average gene essentiality scores (CRISPR‐Cas9 gene knockout scores [CERES]) that reflect cuproptosis gene dependence were calculated in 26 cell lines with GC origin. Drug IC50 value, which was released by Genomics of Drug Sensitivity in Cancer (GDSC1) datasets, was utilized to explore the potential therapeutic association with CSRS scoring [66].

### Construction of the CSRS scoring system

We constructed a CSRS scheme to quantify the relative cuproptosis risk level of individual patients by using principal component analysis (PCA). Specifically, the eigenvalues and eigenvectors of the correlation coefficient matrix on 16 cuproptosis genes were derived and subjected to construct matrix product (PC score). About 79.1% of the variation in PCA analysis is explained by this first five eigenvalues and considered as the number of principal components to retain. Then, the sum of the first five eigenvalues weighted by PC score were served as the cuproptosis signature score. We also adopted a formula to define the CSRS scheme of each GC sample: CSRS = ∑(*E*_i_*PC_*i*_)/E_1:5_, where *E* denotes the Eigen values, PC denotes the principal components, *i* denotes number from 1 to 5. Moreover, the surv‐cutpoint function from R package “survival,” which based on standardized maximally selected log‐rank statistics, was used to determine the optimal point of stratification of high‐risk and low‐risk groups in the CSRS scoring system. The identified optimal cut‐point of CSRS‐score in ACRG was further utilized in TCGA and Yonsei cohort.

### Cell culture

AGS cells and HGC‐27 cells were provided by ATCC. AGS cells and HGC‐27 cells were maintained in RPMI Medium 1640 basic (Gibco; C11875500BT) supplemented with fetal bovine serum (FBS, 10%, PAN; ST30‐3302), penicillin–streptomycin–gentamicin (1%, Solarbio; P1410), and cultured in 95% air and 5% CO_2_ at 37°C.

### Western blot analysis

Observed that the cell density reaches about 90%, we prepared to lyse the cells to extract the protein. The protein concentration was measured by BCA method using a BCA protein assay kit (Solarbio; PC0020). After obtaining the OD value, the final concentration of each sample was calculated according to the concentration formula of standard curve calculation. Prepared a 12% concentration of the gel, we started protein electrophoresis after loading samples. First the protein was pressed in the homogenous bands in the concentrated gel with a constant pressure of 50 V, and then we run the separation gel with 100 V. After electrophoresis, we started to transfer the PVDF membrane, used a constant current of 220 mA, and calculated the transfer time according to 1 min/kDa. Skimmed milk was selected for blocking, and 50 rpm decolorization shaker was used for 1 h. After the PVDF membrane was washed with TBS‐t solution, the primary antibodies targeting FDX1 (Proteintech; 12592‐1‐AP; 19 kDa), LIAS (Proteintech; 11577‐1‐AP, 34–42 kDa), DLD (Proteintech; 16431‐1‐AP; 54 kDa), DLAT (Proteintech; 13426‐1‐AP; 70 kDa), DLST (CST; 5556S; 50 kDa), PDHA1 (Proteintech; 18068‐1‐AP; 43 kDa), PDHB (Proteintech; 14744‐1‐AP; 39 kDa), GCSH (Proteintech; 16726‐1‐AP; 19 kDa), and β‐actin (Proteintech; 20536‐1‐AP; 42 kDa) were added for incubation, and the 50 rpm decolorization shaker was shaken with ice box at 4°C for 14–16 h. After washied the PVDF membrane with TBS‐t solution, we added the corresponding secondary antibody (Proteintech; SA00001‐2) for antibody incubation, and decolorized the shaker at 50 rpm for 1 h. After washed the PVDF membrane with TBS‐t solution, we prepared the developer, added an appropriate amount of developer to each strip, exposed and developed the protein strip, and analyzed the strip with ImageJ1.52v software after storage.

### Cell counting kit‐8 (CCK‐8) assay

Gastric cancer cells were digested and seeded in 96‐well plates with 5000 cells per well. After the cell density reached 90%–95%, the drug was prepared according to the concentration gradient and added into different wells. The specific concentration gradient was: NSC319726 (0, 1.0, 10.0, 100.0, 300.0), thapsigargin (0, 0.1, 1.0, 10.0, 50.0, 100.0), talazoparib (0, 0.1, 1.0, 10.0, 50.0, 100.0). After 24 h of drug treatment, the OD value of the remaining cells at 450 nm was measured by CCK‐8 method with CCK‐8 (DojinDo; CK04), and the survival rate of the cells at different concentrations was calculated and plotted by GraphPad prism 8.

### Plate colony

We inoculated gastric cancer cells into a six‐well plate, with 1000 cells per well. After 24 h of cell growth, we replaced the solution with a drug that measured the concentration of IC50. After 24 h of treatment, we switched to complete culture medium and continued to culture for 8–10 days until the cell colony size was suitable. After washing with the precooled PBS, we fixed it with 4% paraformaldehyde 1 mL for 30 min, dyed it with crystal violet 1 mL for 30 min, and washed it with PBS. Then we took photos with a camera, and counted the number of cell colonies using ImageJ1.52 v software.

### Statistical analyses

The statistical analyses in this study were generated by R‐4.0.2. For quantitative data, statistical significance for normally distributed variables was estimated by student's *t* tests, and non‐normally distributed variables were analyzed by the Wilcoxon rank‐sum test. For comparisons of more than two groups, Kruskal–Wallis tests and one‐way analysis of variance were used as nonparametric and parametric methods, respectively. Chi‐square test and Fisher's exact test were used to analyze contingency tables depending on specific grouping conditions. Kaplan–Meier survival analysis and the Cox proportional hazards model were used to analyze the association between the metabolic transcriptomic pattern and prognosis with the R package “Survminer” (0.4.6). The surv‐cutpoint function from the “survival” package was applied to stratify samples into high and low CSRS‐score subtypes in ACRG cohort. All comparisons were two‐sided with an alpha level of 0.05, and the Benjamini–Hochberg method was applied to control the false discovery rate (FDR) for multiple hypothesis testing.

## AUTHOR CONTRIBUTIONS

Wei Chong and Hao Chen developed the methodology, created models, programmed the computer code, and reviewed the published work. Huicheng Ren performed the experiments, wrote the initial draft, and revised the manuscript. Kang Xu did the experiments, collected the data, and revised the manuscript. Xingyu Zhu, Yuan Liu, Yaodong Sang, Han Li, Jin Liu, and Chunshui Ye collected the data, analyzed the study data, and annotated the study data. Liang Shang and Changqing Jing managed the responsibility for the research activity planning and execution. Leping Li formulated the overarching research goals and aims, gave the financial support, and provided the study materials. All authors have read the final manuscript and approved it for publication.

## CONFLICT OF INTEREST STATEMENT

The authors declare no competing interests.

## Supporting information


**Figure S1**: Correlation and prognostic analysis of 16 cuproptosis‐related genes.
**Figure S2**: Unsupervised clustering description of 16 cuproptosis‐related genes in the gastric cancer cohort.
**Figure S3**: The cuproptosis signature clusters characterized by distinct functional enrichment.
**Figure S4**: The analysis of cuproptosis signature risk scores and the clinical characteristics in gastric cancer.
**Figure S5**: The analysis of single‐nucleotide substitutions and chromosome mutations based on CSRS.

Supporting Information.

## Data Availability

The data that supports the findings of this study are available in the supplementary material of this article. All relevant data and materials within this work are made available in the TCGA (https://cancergenome.nih.gov/; https://www.cbioportal.org/), DepMap (https://depmap.org/portal/download, gene expression [CCLE expression, 21Q1]; Drug sensitivity IC50 [Sanger GDSC1]), and GEO https://www.ncbi.nlm.nih.gov/geo/query/acc.cgi?acc=GSE62254; https://www.ncbi.nlm.nih.gov/geo/query/acc.cgi?acc=GSE84437) database. Essential scripts for implementing cuproptosis signature‐based clustering and risk scoring et al. procedure are available on the Github website (https://github.com/chenhao-qilu/CSRS_STAD). Supplementary materials (figures, tables, scripts, graphical abstract, slides, videos, Chinese translated version and update materials) may be found in the online DOI or iMeta Science http://www.imeta.science/.

## References

[1] Sung, Hyuna , Jacques Ferlay , Rebecca L. Siegel , Mathieu Laversanne , Isabelle Soerjomataram , Ahmedin Jemal , and Freddie Bray . 2021. “Global Cancer Statistics 2020: GLOBOCAN Estimates of Incidence and Mortality Worldwide for 36 Cancers in 185 Countries.” CA: A Cancer Journal for Clinicians 71: 209–249. 10.3322/caac.21660 33538338

[2] Machlowska, Julita , Jacek Baj , Monika Sitarz , Ryszard Maciejewski , and Robert Sitarz . 2020. “Gastric Cancer: Epidemiology, Risk Factors, Classification, Genomic Characteristics and Treatment Strategies.” International Journal of Molecular Sciences 21: 4012. 10.3390/ijms21114012 32512697 PMC7312039

[3] Cristescu, Razvan , Jeeyun Lee , Michael Nebozhyn , Kyoung‐Mee Kim , Jason C. Ting , Swee Seong Wong , Jiangang Liu , et al. 2015. “Molecular Analysis of Gastric Cancer Identifies Subtypes Associated With Distinct Clinical Outcomes.” Nature Medicine 21: 449–456. 10.1038/nm.3850 25894828

[4] Pietrantonio, Filippo , Rosalba Miceli , Alessandra Raimondi , Young Woo Kim , Won Ki Kang , Ruth E. Langley , Yoon Young Choi , et al. 2019. “Individual Patient Data Meta‐Analysis of the Value of Microsatellite Instability as a Biomarker in Gastric Cancer.” Journal of Clinical Oncology 37: 3392–3400. 10.1200/Jco.19.01124 31513484

[5] Sohn, Bo Hwa , Jun‐Eul Hwang , Hee‐Jin Jang , Hyun‐Sung Lee , Sang Cheul Oh , Jae‐Jun Shim , Keun‐Wook Lee , et al. 2017. “Clinical Significance of Four Molecular Subtypes of Gastric Cancer Identified by the Cancer Genome Atlas Project.” Clinical Cancer Research 23: 4441–4449. 10.1158/1078-0432.CCR-16-2211 28747339 PMC5785562

[6] Joshi, Smita S. , and Brian D. Badgwell . 2021. “Current Treatment and Recent Progress in Gastric Cancer.” CA: A Cancer Journal for Clinicians 71: 264–279. 10.3322/caac.21657 33592120 PMC9927927

[7] Chong, Wei , Xingyu Zhu , Huicheng Ren , Chunshui Ye , Kang Xu , Zhe Wang , Shengtao Jia , et al. 2022. “Integrated Multi‐Omics Characterization of KRAS Mutant Colorectal Cancer.” Theranostics 12: 5138–5154. 10.7150/thno.73089 35836817 PMC9274732

[8] Bai, Menglin , Leilei Wu , Mengyu Zhao , Peng Jin , Jingru Liu , Weiqing Wang , Xuetian Gao , et al. 2023. “Integrated Analysis of EGFR Mutated Non‐Small Cell Lung Cancer Reveals Two Distinct Molecular Subtypes.” Clinical and Translational Medicine 13: e1431. 10.1002/ctm2.1431 37830123 PMC10570769

[9] Johnston, Fabian M. , and Michael Beckman . 2019. “Updates on Management of Gastric Cancer.” Current Oncology Reports 21: 67. 10.1007/s11912-019-0820-4 31236716

[10] Bang, Y. J. , G. Giaccone , S. A. Im , D. Y. Oh , T. M. Bauer , J. L. Nordstrom , H. Li , et al. 2017. “First‐in‐Human Phase 1 Study of Margetuximab (MGAH22), an Fc‐Modified Chimeric Monoclonal Antibody, in Patients With HER2‐Positive Advanced Solid Tumors.” Annals of Oncology 28: 855–861. 10.1093/annonc/mdx002 28119295 PMC6246722

[11] Maron, Steven B. , Lindsay Alpert , Heewon A. Kwak , Samantha Lomnicki , Leah Chase , David Xu , Emily O'Day , et al. 2018. “Targeted Therapies for Targeted Populations: Anti‐EGFR Treatment for EGFR‐Amplified Gastroesophageal Adenocarcinoma.” Cancer Discovery 8: 696–713. 10.1158/2159-8290.CD-17-1260 29449271 PMC5984701

[12] Chong, Wei , Liang Shang , Jin Liu , Zhen Fang , Fengying Du , Hao Wu , Yang Liu , et al. 2021. “m(6)A Regulator‐Based Methylation Modification Patterns Characterized by Distinct Tumor Microenvironment Immune Profiles in Colon Cancer.” Theranostics 11: 2201–2217. 10.7150/thno.52717 33500720 PMC7797678

[13] Chen, Hao , Tongchao Zhang , Yuan Zhang , Hao Wu , Zhen Fang , Yang Liu , Yang Chen , et al. 2022. “Deciphering the Tumor Microenvironment Cell‐Infiltrating Landscape Reveals Microenvironment Subtypes and Therapeutic Potentials for Nonsquamous NSCLC.” JCI Insight 7: e152815. 10.1172/jci.insight.152815 35511432 PMC9309061

[14] Ge, Eva J. , Ashley I. Bush , Angela Casini , Paul A. Cobine , Justin R. Cross , Gina M. DeNicola , Q Ping Dou , et al. 2022. “Connecting Copper and Cancer: From Transition Metal Signalling to Metalloplasia.” Nature Reviews Cancer 22: 102–113. 10.1038/s41568-021-00417-2 34764459 PMC8810673

[15] Cen, Dazhi , Daniel Brayton , Babbak Shahandeh , Frank L. Meyskens Jr. , and Patrick J. Farmer . 2004. “Disulfiram Facilitates Intracellular Cu Uptake and Induces Apoptosis in Human Melanoma Cells.” Journal of Medicinal Chemistry 47: 6914–6920. 10.1021/jm049568z 15615540

[16] Chen, Di , Qiuzhi Cindy Cui , Huanjie Yang , and Q Ping Dou . 2006. “Disulfiram, a Clinically used Anti‐Alcoholism Drug and Copper‐Binding Agent, Induces Apoptotic Cell Death in Breast Cancer Cultures and Xenografts via Inhibition of the Proteasome Activity.” Cancer Research 66: 10425–10433. 10.1158/0008-5472.CAN-06-2126 17079463

[17] Brady, Donita C. , Matthew S. Crowe , Danielle N. Greenberg , and Christopher M. Counter . 2017. “Copper Chelation Inhibits BRAF(V600E)‐Driven Melanomagenesis and Counters Resistance to BRAF(V600E) and MEK1/2 Inhibitors.” Cancer Research 77: 6240–6252. 10.1158/0008-5472.CAN-16-1190 28986383 PMC5690876

[18] Tsvetkov, Peter , Shannon Coy , Boryana Petrova , Margaret Dreishpoon , Ana Verma , Mai Abdusamad , Jordan Rossen , et al. 2022. “Copper Induces Cell Death by Targeting Lipoylated TCA Cycle Proteins.” Science 375: 1254–1261. 10.1126/science.abf0529 35298263 PMC9273333

[19] Davis, Caroline I. , Xingxing Gu , Ryan M. Kiefer , Martina Ralle , Terence P. Gade , and Donita C. Brady . 2020. “Altered Copper Homeostasis Underlies Sensitivity of Hepatocellular Carcinoma to Copper Chelation.” Metallomics 12: 1995–2008. 10.1039/d0mt00156b 33146201 PMC8315290

[20] Tsang, Tiffany , Jessica M. Posimo , Andrea A. Gudiel , Michelle Cicchini , David M. Feldser , and Donita C. Brady . 2020. “Copper is an Essential Regulator of the Autophagic Kinases ULK1/2 to Drive Lung Adenocarcinoma.” Nature Cell Biology 22: 412–424. 10.1038/s41556-020-0481-4 32203415 PMC7610258

[21] Cui, Liyang , Arvin M. Gouw , Edward L. LaGory , Shenghao Guo , Nabeel Attarwala , Yao Tang , Ji Qi , et al. 2021. “Mitochondrial Copper Depletion Suppresses Triple‐Negative Breast Cancer in Mice.” Nature Biotechnology 39: 357–367. 10.1038/s41587-020-0707-9 PMC7956242 33077961

[22] Zhang, Zhen , Xiangyang Zeng , Yinghua Wu , Yang Liu , Xi Zhang , and Zewen Song . 2022. “Cuproptosis‐Related Risk Score Predicts Prognosis and Characterizes the Tumor Microenvironment in Hepatocellular Carcinoma.” Frontiers in Immunology 13: 925618. 10.3389/fimmu.2022.925618 35898502 PMC9311491

[23] Sha, Shengnan , Luyi Si , Xinrui Wu , Yuanbiao Chen , Hui Xiong , Ying Xu , Wangrui Liu , et al. 2022. “Prognostic Analysis of Cuproptosis‐Related Gene in Triple‐Negative Breast Cancer.” Frontiers in Immunology 13: 922780. 10.3389/fimmu.2022.922780 35979353 PMC9376234

[24] Yang, Mingyi , Haishi Zheng , Ke Xu , Qiling Yuan , Yirixaiti Aihaiti , Yongsong Cai , and Peng Xu . 2022. “A Novel Signature to Guide Osteosarcoma Prognosis and Immune Microenvironment: Cuproptosis‐Related lncRNA.” Frontiers in Immunology 13: 919231. 10.3389/fimmu.2022.919231 35967366 PMC9373797

[25] Lv, Haozhen , Xiao Liu , Xuanhao Zeng , Yating Liu , Canjing Zhang , Qi Zhang , and Jinhua Xu . 2022. “Comprehensive Analysis of Cuproptosis‐Related Genes in Immune Infiltration and Prognosis in Melanoma.” Frontiers in Pharmacology 13: 930041. 10.3389/fphar.2022.930041 35837286 PMC9273972

[26] Ma, Chao , Feng Li , Zhanfeng He , Song Zhao , Yang Yang , and Zhuoyu Gu . 2023. “Prognosis and Personalized Treatment Prediction in Lung Adenocarcinoma: an In Silico and In Vitro Strategy Adopting Cuproptosis Related lncRNA Towards Precision Oncology.” Frontiers in Pharmacology 14: 1113808. 10.3389/fphar.2023.1113808 36874011 PMC9975170

[27] Pan, Shize , Congkuan Song , Heng Meng , Ning Li , Donghang Li , Bo Hao , Zilong Lu , and Qing Geng . 2022. “Identification of Cuproptosis‐Related Subtypes in Lung Adenocarcinoma and its Potential Significance.” Frontiers in Pharmacology 13: 934722. 10.3389/fphar.2022.934722 36263125 PMC9573969

[28] Xiaona, Xie , Qianzi Liu , Xuehua Zhou , Rongtao Liang , Shengbo Yang , Min Xu , Haiyang Zhao , et al. 2023. “Comprehensive Analysis of Cuproptosis‐Related Genes in Immune Infiltration and Prognosis in Lung Adenocarcinoma.” Computers in Biology and Medicine 158: 106831. 10.1016/j.compbiomed.2023.106831 37037146

[29] Liu, Xianglong , Bo Sun , Yiyang Yao , Linying Lai , Xueyuan Wang , Jie Xiong , Xiaoan Zhang , and Jie Jiang . 2022. “Identification of Copper Metabolism and Cuproptosis‐Related Subtypes for Predicting Prognosis Tumor Microenvironment and Drug Candidates in Hepatocellular Carcinoma.” Frontiers in Immunology 13: 996308. 10.3389/fimmu.2022.996308 36275743 PMC9582144

[30] Ma, Wei , Lingyuan Zhu , Shushu Song , Bo Liu , and Jianxin Gu . 2022. “Identification and Validation of Glycosyltransferases Correlated With Cuproptosis as a Prognostic Model for Colon Adenocarcinoma.” Cells 11: 3728. 10.3390/cells11233728 36496988 PMC9737711

[31] Yan, Cheng , Yandie Niu , Liukai Ma , Lifang Tian , and Jiahao Ma . 2022. “System Analysis Based on the Cuproptosis‐Related Genes Identifies LIPT1 as a Novel Therapy Target for Liver Hepatocellular Carcinoma.” Journal of Translational Medicine 20: 452. 10.1186/s12967-022-03630-1 36195876 PMC9531858

[32] Lee, Song Yi , Ji‐Hye Seo , Sungyun Kim , ChaeRim Hwang , Da In Jeong , JiHye Park , Mingyu Yang , Ji Won Huh , and Hyun‐Jong Cho . 2023. “Cuproptosis‐Inducible Chemotherapeutic/Cascade Catalytic Reactor System for Combating With Breast Cancer.” Small 19: e2301402. 10.1002/smll.202301402 37162448

[33] Ning, Shipeng , Meng Lyu , Daoming Zhu , Jacky W. Y. Lam , Qinqin Huang , Tianfu Zhang , and Ben Zhong Tang . 2023. “Type‐I AIE Photosensitizer Loaded Biomimetic System Boosting Cuproptosis to Inhibit Breast Cancer Metastasis and Rechallenge.” ACS Nano 17: 10206–10217. 10.1021/acsnano.3c00326 37183977

[34] Zeng, Dongqiang , Zilan Ye , Rongfang Shen , Guangchuang Yu , Jiani Wu , Yi Xiong , Rui Zhou , et al. 2021. “IOBR: Multi‐Omics Immuno‐Oncology Biological Research to Decode Tumor Microenvironment and Signatures.” Frontiers in Immunology 12: 687975. 10.3389/fimmu.2021.687975 34276676 PMC8283787

[35] Tang, Daolin , Xin Chen , and Guido Kroemer . 2022. “Cuproptosis: a Copper‐Triggered Modality of Mitochondrial Cell Death.” Cell Research 32: 417–418. 10.1038/s41422-022-00653-7 35354936 PMC9061796

[36] Meng, Jialin , Aimin Jiang , Xiaofan Lu , Di Gu , Qintao Ge , Suwen Bai , Yundong Zhou , et al. 2023. “Multiomics Characterization and Verification of Clear Cell Renal Cell Carcinoma Molecular Subtypes to Guide Precise Chemotherapy and Immunotherapy.” iMeta 2: e147. 10.1002/imt2.147 38868222 PMC10989995

[37] Chen, Guoming , Dongqiang Luo , Xiangjun Qi , Danyun Li , Jiyuan Zheng , Yang Luo , Cheng Zhang , et al. 2023. “Characterization of Cuproptosis in Gastric Cancer and Relationship With Clinical and Drug Reactions.” Frontiers in Cell and Developmental Biology 11: 1172895. 10.3389/fcell.2023.1172895 37351275 PMC10283039

[38] Jiang, Lin , Junzuo Liao , and Yunwei Han . 2023. “Study on the Role and Pharmacology of Cuproptosis in Gastric Cancer.” Frontiers in Oncology 13: 1145446. 10.3389/fonc.2023.1145446 37007099 PMC10063964

[39] Li, Jiazheng , Can Kong , Wei Song , and Tao Fu . 2023. “Identification of Cuproptosis‐Related Subtypes, Establishment of a Prognostic Signature and Characterization of the Tumor Microenvironment in Gastric Cancer.” International Journal of General Medicine 16: 1631–1652. 10.2147/IJGM.S404847 37168531 PMC10164657

[40] Hu, Yongli , Yan Du , Zhisheng Qiu , Pengwei Bai , Zhaozhao Bai , Chenglou Zhu , Junhong Wang , Tong Liang , and Mingxu Da . 2023. “Construction of a Cuproptosis‐Related Gene Signature for Predicting Prognosis in Gastric Cancer.” Biochemical Genetics 62: 40–58. 10.1007/s10528-023-10406-9 37243753

[41] Liu, Hui‐Yun , Hong‐Sheng Zhang , Min‐Yao Liu , Hong‐Ming Li , Xin‐Yu Wang , and Miao Wang . 2021. “GLS1 Depletion Inhibited Colorectal Cancer Proliferation and Migration via redox/Nrf2/autophagy‐dependent Pathway.” Archives of Biochemistry And Biophysics 708: 108964. 10.1016/j.abb.2021.108964 34119480

[42] Wang, Lizong , Yi Cao , Wei Guo , and Jingyun Xu . 2023. “High Expression of Cuproptosis‐Related Gene FDX1 in Relation to Good Prognosis and Immune Cells Infiltration in Colon Adenocarcinoma (COAD).” Journal of Cancer Research and Clinical Oncology 149: 15–24. 10.1007/s00432-022-04382-7 36173462 PMC9889456

[43] Barresi, Vincenza , Angela Trovato‐Salinaro , Giorgia Spampinato , Nicolò Musso , Sergio Castorina , Enrico Rizzarelli , and Daniele Filippo Condorelli . 2016. “Transcriptome Analysis of Copper Homeostasis Genes Reveals Coordinated Upregulation of SLC31A1,SCO1, and COX11 in Colorectal Cancer.” FEBS Open Bio 6: 794–806. 10.1002/2211-5463.12060 PMC4971835 27516958

[44] Martinez‐Balibrea, Eva , Anna Martínez‐Cardús , Eva Musulén , Alba Ginés , José Luis Manzano , Enrique Aranda , Carmen Plasencia , Nouri Neamati , and Albert Abad . 2009. “Increased Levels of Copper Efflux Transporter ATP7B are Associated With Poor Outcome in Colorectal Cancer Patients Receiving Oxaliplatin‐Based Chemotherapy.” International Journal of Cancer 124: 2905–2910. 10.1002/ijc.24273 19296535

[45] Song, Liying , Rong Zeng , Keda Yang , Wei Liu , Zhijie Xu , and Fanhua Kang . 2023. “The Biological Significance of Cuproptosis‐Key Gene MTF1 in Pan‐Cancer and its Inhibitory Effects on ROS‐Mediated Cell Death of Liver Hepatocellular Carcinoma.” Discover Oncology 14: 113. 10.1007/s12672-023-00738-8 37380924 PMC10307746

[46] Bedard, Philippe L. , David M. Hyman , Matthew S. Davids , and Lillian L. Siu . 2020. “Small Molecules, Big Impact: 20 Years of Targeted Therapy in Oncology.” The Lancet 395: 1078–1088. 10.1016/S0140-6736(20)30164-1 32222192

[47] Nakamura, Yoshiaki , Akihito Kawazoe , Florian Lordick , Yelena Y. Janjigian , and Kohei Shitara . 2021. “Biomarker‐Targeted Therapies for Advanced‐Stage Gastric and Gastro‐Oesophageal Junction Cancers: an Emerging Paradigm.” Nature Reviews Clinical Oncology 18: 473–487. 10.1038/s41571-021-00492-2 33790428

[48] Xia, Jie , Zhangjuan Xie , Gengming Niu , Zhou Lu , Zhiqiang Wang , Yun Xing , Jun Ren , et al. 2023. “Single‐Cell Landscape and Clinical Outcomes of Infiltrating B Cells in Colorectal Cancer.” Immunology 168: 135–151. 10.1111/imm.13568 36082430

[49] Huang, Kaimei , Binghu Lin , Jinyang Liu , Yankun Liu , Jingwu Li , Geng Tian , and Jialiang Yang . 2022. “Predicting Colorectal Cancer Tumor Mutational Burden From Histopathological Images and Clinical Information Using Multi‐Modal Deep Learning.” Bioinformatics 38: 5108–5115. 10.1093/bioinformatics/btac641 36130268

[50] Luo, Qianxin , Dianke Chen , Xinjuan Fan , Xinhui Fu , Tenghui Ma , and Daici Chen . 2020. “KRAS and PIK3CA Bi‐Mutations Predict a Poor Prognosis in Colorectal Cancer Patients: A Single‐Site Report.” Translational Oncology 13: 100874. 10.1016/j.tranon.2020.100874 32947236 PMC7502368

[51] Shanbhag, Vinit , Kimberly Jasmer‐McDonald , Sha Zhu , Adam L. Martin , Nikita Gudekar , Aslam Khan , Erik Ladomersky , et al. 2019. “ATP7A Delivers Copper to the Lysyl Oxidase Family of Enzymes and Promotes Tumorigenesis and Metastasis.” Proceedings of the National Academy of Sciences 116: 6836–6841. 10.1073/pnas.1817473116 PMC6452744 30890638

[52] Ash, Dipankar , Varadarajan Sudhahar , Seock‐Won Youn , Mustafa Nazir Okur , Archita Das , John P. O'Bryan , Maggie McMenamin , et al. 2021. “The P‐Type ATPase Transporter ATP7A Promotes Angiogenesis by Limiting Autophagic Degradation of VEGFR2.” Nature Communications 12: 3091. 10.1038/s41467-021-23408-1 PMC8149886 34035268

[53] Goh, Wen Quan Jonathan , Ghim Siong Ow , Vladimir A. Kuznetsov , Shirly Chong , and Yoon Pin Lim . 2015. “DLAT Subunit of the Pyruvate Dehydrogenase Complex is Upregulated in Gastric Cancer‐Implications in Cancer Therapy.” American Journal of Translational Research 7: 1140–1151. https://www.ncbi.nlm.nih.gov/pubmed/26279757 26279757 PMC4532746

[54] Chen, Qianqian , Yiling Wang , Lin Yang , Liyuan Sun , Yuxin Wen , Yongyi Huang , Kaiping Gao , et al 2022. “PM2.5 Promotes NSCLC Carcinogenesis Through Translationally and Transcriptionally Activating DLAT‐Mediated Glycolysis Reprograming.” Journal of Experimental & Clinical Cancer Research 41: 229. 10.1186/s13046-022-02437-8 35869499 PMC9308224

[55] Zhang, Jin , Xiangmudong Kong , Yanhong Zhang , Wenqiang Sun , Jian Wang , Mingyi Chen , and Xinbin Chen . 2020. “FDXR Regulates TP73 Tumor Suppressor via IRP2 To Modulate Aging and Tumor Suppression.” The Journal of Pathology 251: 284–296. 10.1002/path.5451 32304229 PMC7748393

[56] Zhang, Yanhong , Yingjuan Qian , Jin Zhang , Wensheng Yan , Yong‐Sam Jung , Mingyi Chen , Eric Huang , et al 2017. “Ferredoxin Reductase is Critical for p53‐Dependent Tumor Suppression via Iron Regulatory Protein 2.” Genes & Development 31: 1243–1256. 10.1101/gad.299388.117 28747430 PMC5558926

[57] Zhang, Zeyu , Yarui Ma , Xiaolei Guo , Yingxi Du , Qing Zhu , Xiaobing Wang , and Changzhu Duan . 2021. “FDX1 Can Impact the Prognosis and Mediate the Metabolism of Lung Adenocarcinoma.” Frontiers in Pharmacology 12: 749134. 10.3389/fphar.2021.749134 34690780 PMC8531531

[58] Zhang, Chi , Yuanxiao Zeng , Xiuchen Guo , Hangjing Shen , Jianhao Zhang , Kaikai Wang , Mengmeng Ji , and Shengwei Huang . 2022. “Pan‐Cancer Analyses Confirmed the Cuproptosis‐Related Gene FDX1 as an Immunotherapy Predictor and Prognostic Biomarker.” Frontiers in Genetics 13: 923737. 10.3389/fgene.2022.923737 35991547 PMC9388757

[59] Zhao, Lin‐Ping , Rongrong Zheng , Renjiang Kong , Chuyu Huang , Xiaona Rao , Ni Yang , A‐Li Chen , et al. 2022. “Self‐Delivery Ternary Bioregulators for Photodynamic Amplified Immunotherapy by Tumor Microenvironment Reprogramming.” ACS Nano 16: 1182–1197. 10.1021/acsnano.1c08978 35023720

[60] Fujiki, Kota , Hisako Inamura , Takeshi Sugaya , and Masato Matsuoka . 2019. “Blockade of ALK4/5 Signaling Suppresses Cadmium‐ and Erastin‐Induced Cell Death in Renal Proximal Tubular Epithelial Cells via Distinct Signaling Mechanisms.” Cell Death & Differentiation 26: 2371–2385. 10.1038/s41418-019-0307-8 30804470 PMC6889492

[61] Wang, Congcong , Tao Li , Shusheng Tang , Dongxu Zhao , Chaoming Zhang , Shen Zhang , Sijun Deng , Yan Zhou , and Xilong Xiao . 2016. “Thapsigargin Induces Apoptosis When Autophagy is Inhibited in HepG2 Cells and Both Processes Are Regulated By ROS‐dependent Pathway.” Environmental Toxicology and Pharmacology 41: 167–179. 10.1016/j.etap.2015.11.020 26708201

[62] Shimada, Kenichi , Eduard Reznik , Michael E. Stokes , Lakshmi Krishnamoorthy , Pieter H. Bos , Yuyu Song , Christine E. Quartararo , et al. 2018. “Copper‐Binding Small Molecule Induces Oxidative Stress and Cell‐Cycle Arrest in Glioblastoma‐Patient‐Derived Cells.” Cell Chemical Biology 25: 585–594.e7. 10.1016/j.chembiol.2018.02.010 29576531 PMC5959763

[63] Taylor, Alison M. , Juliann Shih , Gavin Ha , Galen F. Gao , Xiaoyang Zhang , Ashton C. Berger , Steven E. Schumacher , et al 2018. “Genomic and Functional Approaches to Understanding Cancer Aneuploidy.” Cancer Cell 33: 676–689.e3. 10.1016/j.ccell.2018.03.007 29622463 PMC6028190

[64] Barbie, David A. , Pablo Tamayo , Jesse S. Boehm , So Young Kim , Susan E. Moody , Ian F. Dunn , Anna C. Schinzel , et al. 2009. “Systematic RNA Interference Reveals That Oncogenic KRAS‐driven Cancers Require TBK1.” Nature 462: 108–112. 10.1038/nature08460 19847166 PMC2783335

[65] Charoentong, Pornpimol ,, Francesca Finotello , Mihaela Angelova , Clemens Mayer , Mirjana Efremova , Dietmar Rieder , Hubert Hackl , and Zlatko Trajanoski . 2017. “Pan‐Cancer Immunogenomic Analyses Reveal Genotype‐Immunophenotype Relationships and Predictors of Response to Checkpoint Blockade.” Cell Reports 18: 248–262. 10.1016/j.celrep.2016.12.019 28052254

[66] Shen, Weitao , Ziguang Song , Xiao Zhong , Mei Huang , Danting Shen , Pingping Gao , and Xiaoqian Qian , et al. 2022“Sangerbox: A Comprehensive, Interaction‐Friendly Clinical Bioinformatics Analysis Platform.” iMeta 1(3), e36. 10.1002/imt2.36 38868713 PMC10989974

