# Supplementary material for: Clinical features and molecular landscape of cuproptosis signature‐related molecular subtype in gastric cancer
Source: Imeta. 2024 Apr 5;3(3):e190. doi: 10.1002/imt2.190 (PMC11183172; doi:10.1002/imt2.190)
Supplement: Supplementary file 1 — Figure S1: Correlation and prognostic analysis of 16 cuproptosis‐related genes. Figure S2: Unsupervised clustering description of 16 cuproptosis‐related genes in the gastric cancer cohort. Figure S3: The cuproptosis signature clusters characterized by distinct functional enrichment. Figure S4: The analysis of cuproptosis signature risk scores and the clinical characteristics in gastric cancer. Figure S5: The analysis of single‐nucleotide substitutions and chromosome mutations based on CSRS. [file IMT2-3-e190-s001.docx]

**Supporting information to** **Clinical Features and Molecular Landscape of Cuproptosis Signature Related Molecular Subtype in Gastric Cancer**

**Running title: Clinical and Molecular Characteristics of Cuproptosis Signature Subtypes in GC**

Wei Chong^1,2#*^, Huicheng Ren^3#^, Hao Chen^4#^, Kang Xu^1,2#^, Xingyu Zhu^1,2^, Yuan Liu^2^, Yaodong Sang^1,2^, Han Li^5^, Jin Liu^6^, Chunshui Ye^2^, Liang Shang^1,2*^, Changqing Jing^1,2*^, Leping Li^1,2*^

^1^Department of Gastrointestinal Surgery, Shandong Provincial Hospital Affiliated to Shandong First Medical University, Jinan, 250021, China,

^2^Key Laboratory of Engineering of Shandong Province, Shandong Provincial Hospital, Medical Science and Technology Innovation Center, Shandong First Medical University & Shandong Academy of Medical Sciences, Jinan, 250021, China,

^3^Department of Gastrointestinal Surgery, Zibo Central Hospital, Zibo, 250036, China,

^4^Clinical Research Center of Shandong University, Clinical Epidemiology Unit, Qilu Hospital of Shandong University, Jinan, 250021, China,

^5^Department of Gastroenterological Surgery, The First Affiliated Hospital of Shandong First Medical University, Jinan, 250021, China,

^6^Department of Gastroenterology, Shandong Provincial Hospital Affiliated to Shandong First Medical University, Jinan, 250021, China.

^#^These authors contributed equally to this work.

^*^Correspondence: [chongwei@sdfmu.edu.cn](mailto:chongwei@sdfmu.edu.cn) (Wei Chong)**;** [lileping@medmail.com.cn](mailto:lileping@medmail.com.cn) (Leping Li)**;** [jingchangqing@sdfmu.edu.cn](mailto:jingchangqing@sdfmu.edu.cn) (Changqing Jing)**;** [docshang@163.com](mailto:docshang@163.com) (Liang Shang)


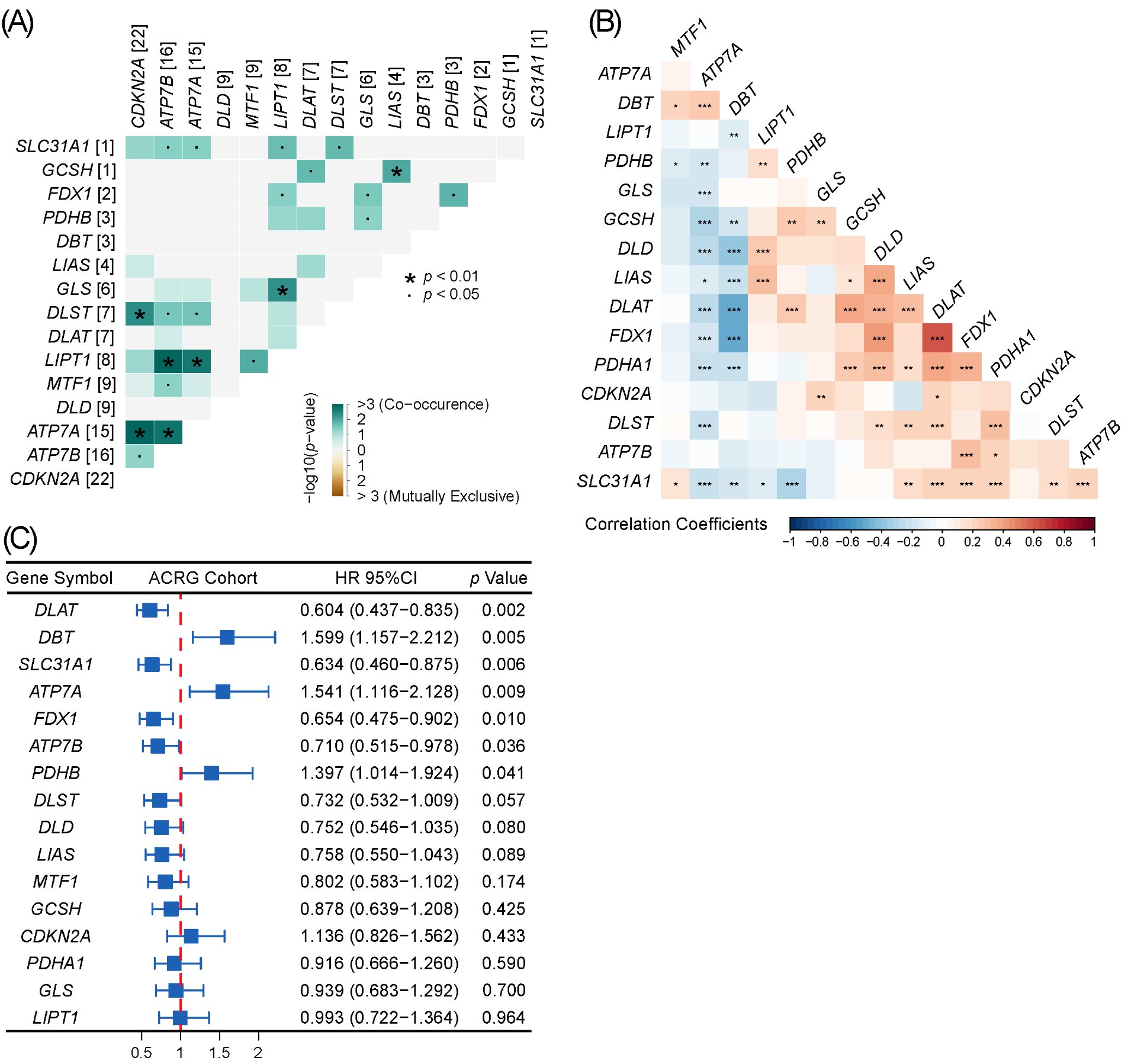


**Figure S1. Correlation and prognostic analysis of 16 cuproptosis-related genes.**

(A) The mutation co-occurrence and exclusion analysis for 16 cuproptosis-related genes. Gene mutation with co-occurrence were colored by green; gene mutation with exclusion were colored by brown. (B) Correlations between cuproptosis-related genes using Spearman analysis. The negative correlation was marked with blue and positive correlation with red. (C) Subgroup analysis estimating clinical prognostic significance of cuproptosis-related genes by univariate Cox regression. The length of the horizontal line represents the 95% confidence interval for each group. The vertical dotted line represented the hazard ratio (HR) of all patients. The vertical solid line represented HR = 1. Hazard ratio >1 represented risk factor for survival and hazard ratio < 1 represented protective factor for survival.


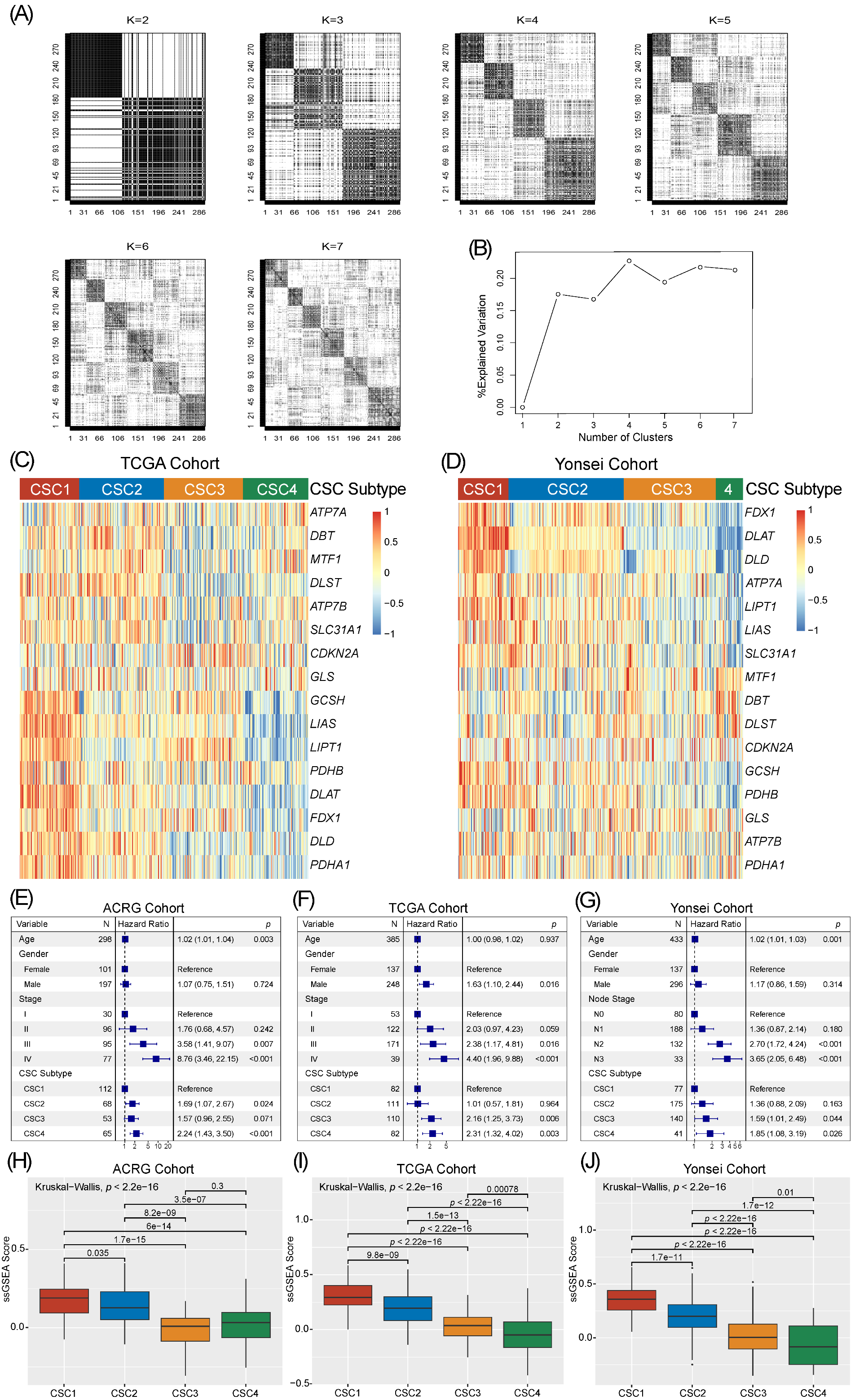


**Figure S2. Unsupervised clustering description of 16 cuproptosis-related genes in the gastric cancer cohort.**

(A) Heatmap representation of iClusterPlus clustering for cuproptosis-related genes in GC dataset with cluster numbers from 2 to 7. (B) The number of 4 clusters accounted for the maximum value of the explained variation ranges from 2 to 7. (C-D) Heatmap shows the scaled expression of 16 cuproptosis-related genes in TCGA (C) and Yonsei (D) in distinct cuproptosis signature clusters. Red represented the high enrichment of cuproptosis-related genes and blue represented the low enrichment. (E-G) Forest plot representation of multivariate Cox model depicted association between clusters and overall survival (OS) after being adjusted for age, sex, and stage in ACRG (E), TCGA (F) and Yonsei (G) datasets. (H-J) Distribution of cuproptosis scores among CSCs in ACRG (H), TCGA (I) and Yonsei (J) datasets. Square data markers indicate estimated hazard ratios (HRs) and the length of the horizontal line represented the 95% confidence interval for each variable.


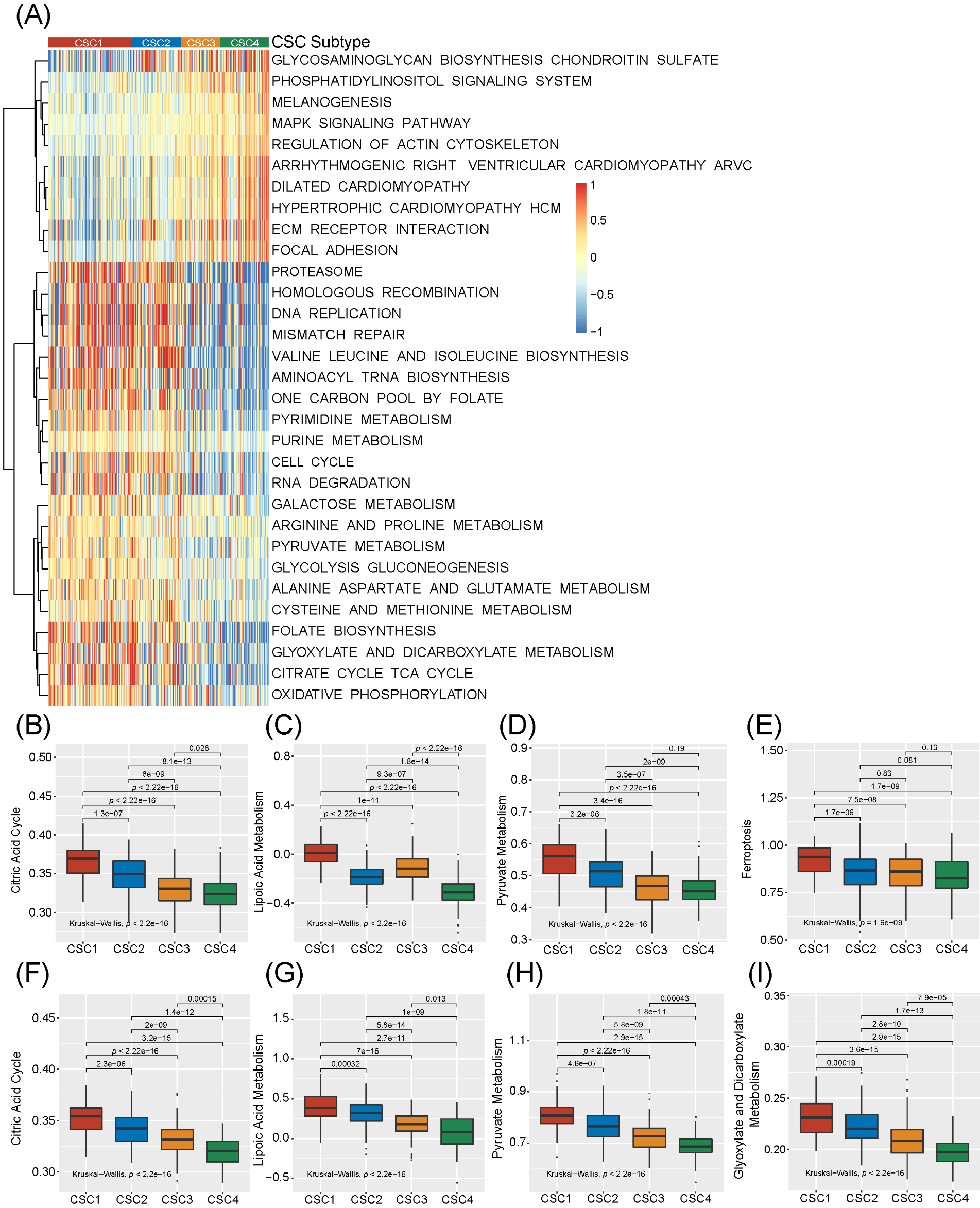


**Figure S3. The cuproptosis signature clusters characterized by distinct functional enrichment.**

(A) Heatmap shows the enrichment score of KEGG signal pathways in the 4 different cuproptosis signature clusters. (B-I) Distribution of cuproptosis signatures, including Citric acid cycle, Ferroptosis, Lipoic acid metabolism, Pyruvate metabolism and glyoxylate and dicarboxylate metabolism among CSC1, CSC2, CSC3 and CSC4 subgroups in TCGA (B-E) and Yonsei cohort (F-I).


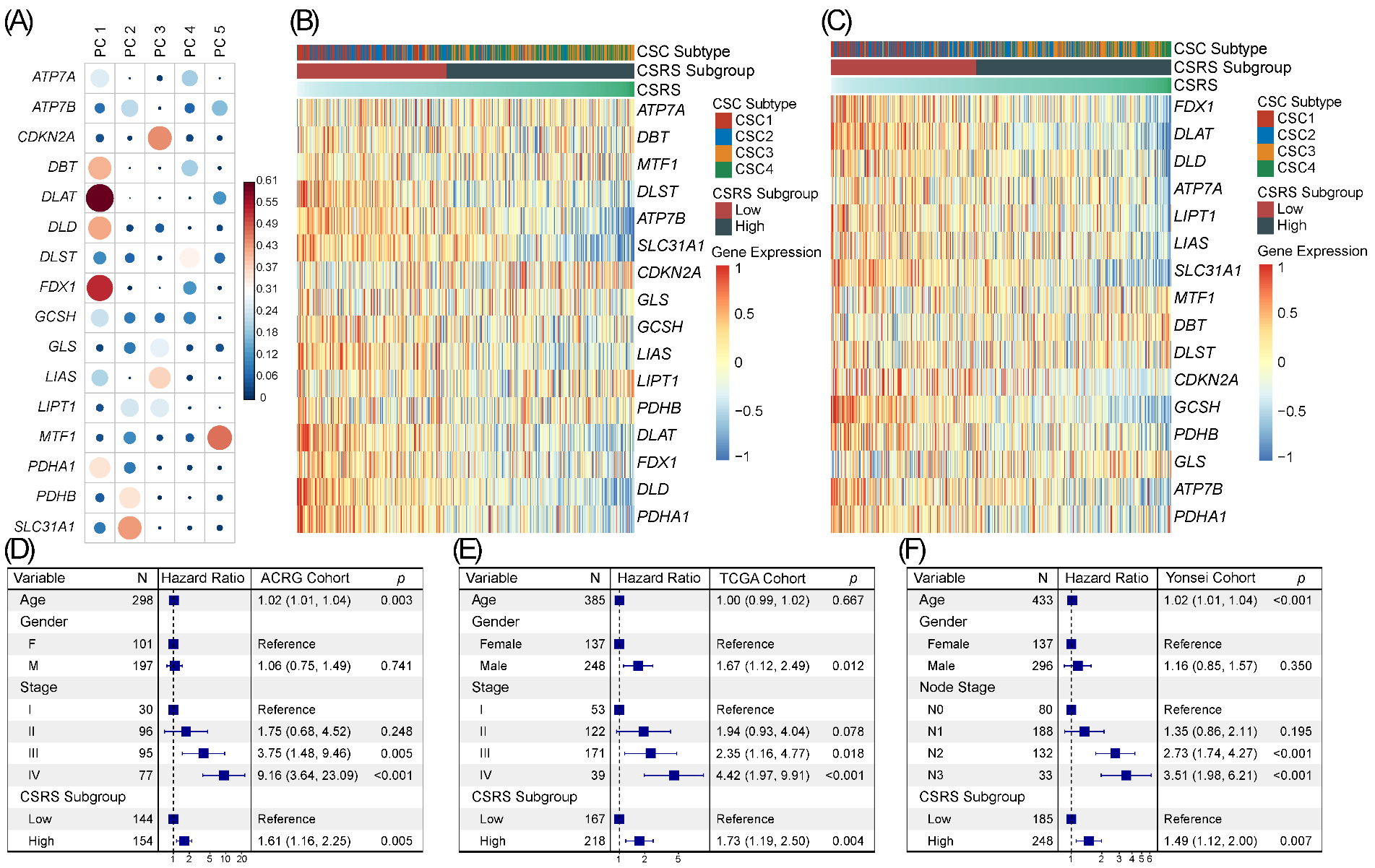


**Figure S4. The analysis of cuproptosis signature risk scores and the clinical characteristics in gastric cancer.**

(A)The contribution of 16 cuproptosis-related genes to first 5 principal components in PCA analysis. (B-C) Heatmap shows the expression level of the 16 cuproptosis-related genes between 4 different cuproptosis signature clusters (CSC) and 2 cuproptosis signature risk scores (CSRS) subgroups. Each column represented single patients. (D-F) Forest plot representation of multivariate Cox model depicted association between CSRS subgroup and overall survival (OS) after being adjusted for age, sex, and stage in ACRG (D), TCGA (E) and Yonsei (F) datasets. Square data markers indicate estimated hazard ratios (HRs) and the length of the horizontal line represents the 95% confidence interval for each variable.


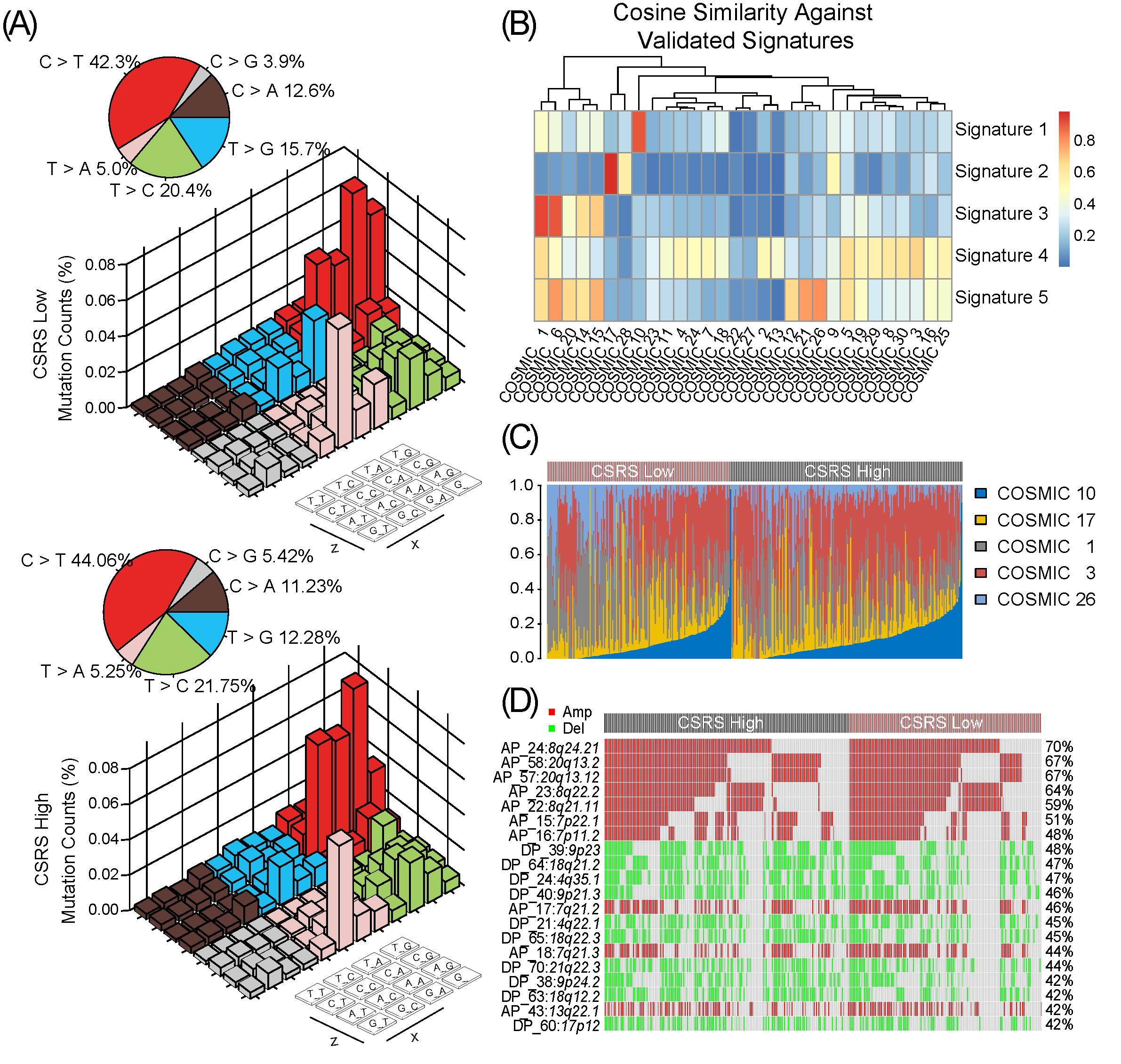


**Figure S5. The analysis of single-nucleotide substitutions and chromosome mutations based on CSRS.**

(A) Lego plot representation of 96 nucleotide mutation patterns of CSRS low-score and high-score samples in TCGA gastric cancer. Single-nucleotide substitutions were divided into six categories with 16 surrounding flanking bases. The pie chart at upper left showed the proportion of six major categories of nucleotide variation. (B) Five mutational signatures were extracted from the genomic data and annotated against the COSMIC V3 nomenclature by cosine similarity analysis. (C) Scaled mutational counts of five signatures between two CSRS subgroups. (D) Distribution of top-ranked CNV sites in two CSRS subgroups.
